# *In Vivo* and *In Vitro* Analyses of Novel Peptidomimetic Disruptors for the Serotonin 5-HT_2C_ Receptor Interaction With Phosphatase and Tensin Homolog

**DOI:** 10.3389/fphar.2019.00907

**Published:** 2019-08-23

**Authors:** Claudia A. Soto, Huang-Chi Du, Robert G. Fox, Taegyun Yang, James Hooson, Noelle C. Anastasio, Scott R. Gilbertson, Kathryn A. Cunningham

**Affiliations:** ^1^Center for Addiction Research and Department of Pharmacology and Toxicology, University of Texas Medical Branch, Galveston, TX, United States; ^2^Department of Chemistry, University of Houston, Houston, TX, United States

**Keywords:** serotonin 5-HT2C receptor, protein–protein interactions, protein phosphatase and tensin homolog, peptidomimetics, drug discrimination

## Abstract

Hypofunction of the serotonin (5-HT) 5-HT_2C_ receptor (5-HT_2C_R) has been implicated in a variety of disorders including substance use disorders. As such, approaches to enhance 5-HT_2C_R signaling display therapeutic potential. In the present study, we show that disruption of the 5-HT_2C_R interaction with the protein phosphatase and tensin homolog (PTEN) *via* peptidomimetics enhances 5-HT_2C_R-mediating signaling *in vitro* and potentiates selective 5-HT_2C_R agonists in behavioral rodent models. Overall, the present study provides further evidence that 5-HT_2C_R activity can be modulated through an allosteric protein–protein interaction. This work provides the groundwork for the continued exploration of protein–protein interactions that can allosterically modulate this critical receptor and other important G protein-coupled receptors (GPCRs) for new therapeutic development through mechanisms that may display clinical utility.

## Introduction

The serotonin (5-HT) 5-HT_2C_ receptor (5-HT_2C_R) is a G protein-coupled receptor (GPCR) that is engaged in normal physiology (e.g., appetite) (Heisler et al., 2003), while 5-HT_2C_R dysfunction is implicated in multiple pathological disorders (e.g., anxiety, depression, obesity, substance use disorders) (Miller, 2005; Howell and Cunningham, 2015). In particular, genetic, biochemical, and pharmacological analyses have implicated 5-HT_2C_R hypofunction as a regulator of behaviors (for review, see Cunningham and Anastasio, 2014; Howell and Cunningham, 2015; Di Giovanni and De Deurwaerdere, 2016). For example, selective 5-HT_2C_R agonists have shown efficacy and potency to reduce food consumption (Gustafson et al., 2013) and impulsivity (Navarra et al., 2008; Anastasio et al., 2013) and the reinforcing (Cunningham et al., 2011; Higgins et al., 2012; Kasper et al., 2013; Neelakantan et al., 2017) and subjective effects (Callahan and Cunningham, 1995; Higgins et al., 2012) of drugs of abuse (i.e., cocaine, ethanol, methamphetamine, nicotine, oxycodone), among other behaviors.

**Figure 1:**
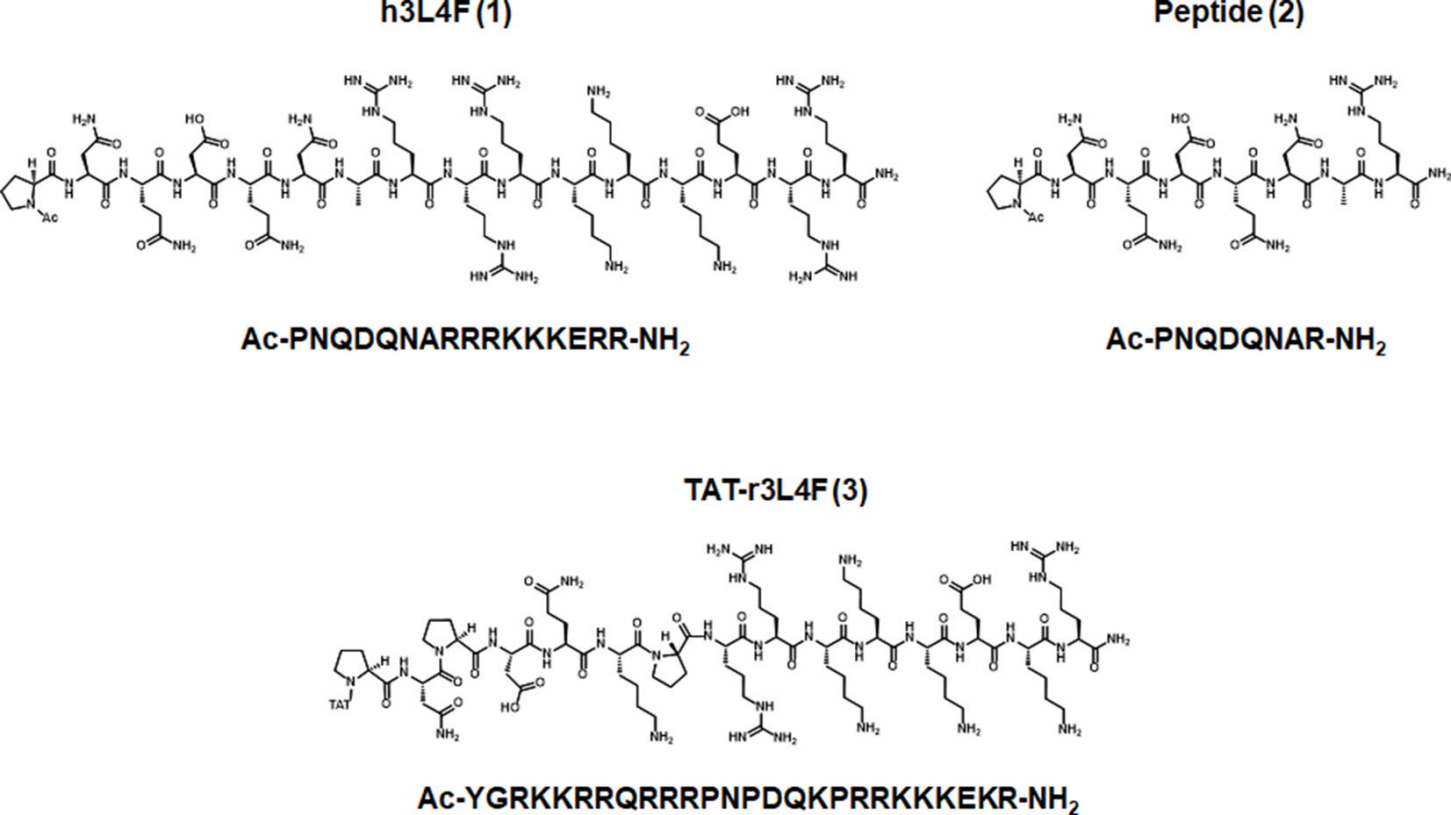
Parent peptides h3L4F (1), 2, and TAT-r3L4F (3), modified from Anastasio et al., 2013.

Agonist binding to the 5-HT_2C_R results in dynamic changes in receptor conformation and induction of a variety of intracellular signaling pathways (Berg et al., 1994; McGrew et al., 2002; Werry et al., 2005; Labasque et al., 2008). Best characterized is coupling of the 5-HT_2C_R to Gα_q/11_ proteins to activate phospholipase C_β_ (PLC_β_), resulting in increased intracellular calcium (Ca*_i_* ^2+^) release, among various other signaling outcomes (Millan et al., 2008). One approach to selectively target the 5-HT_2C_R signaling is through protein–protein interactions that are selective for 5-HT_2C_R over the homologous 5-HT_2A_R and 5-HT_2B_R. In that regard, one protein–protein interaction of interest is the interaction between the 5-HT_2C_R and accessory protein phosphatase and tensin homolog (PTEN) that occurs at the third intracellular loop of the 5-HT_2C_R (Ji et al., 2006; Anastasio et al., 2013). PTEN is a dual phosphatase that contains distinct lipid and protein phosphatase activities and is involved in the suppression of cell proliferation pathways through its lipid phosphatase activity (Ning et al., 2004). Disruption of the 5-HT_2C_R-PTEN complex enhances selective 5-HT_2C_R agonist-induced effects in both cellular and rodent models (Ji et al., 2006; Anastasio et al., 2013). Previous studies employed a 16 amino acid peptide homologous to a fragment of the third intracellular loop of the human 5-HT_2C_R [h3L4F (**1**); **Figure 1**; Ac-PNQDQNARRRKKKERR-NH_2_; Pro280-Arg295] and its shorter version (peptide **2**; **Figure 1**; Ac-PNQDQNAR-NH_2_; Pro280-Arg287) to disrupt the 5-HT_2C_R-PTEN complex (Ji et al., 2006; Anastasio et al., 2013). Peptides **1** and **2** enhance 5-HT_2C_R agonist-mediated Ca*_i_* ^2+^ release in Chinese hamster ovary (CHO) cells stably expressing the human 5-HT_2C_R (h5-HT_2C_R-CHO) (Anastasio et al., 2013). The initial *in vivo* profile of 5-HT_2C_R-PTEN complex disruption in rats was assessed using a peptide homologous to an analogous fragment of the rat 5-HT_2C_R third intracellular loop (r3L4F; Ac-PNPDQKPRRKKKEKR-NH_2_) conjugated to a short cell penetrant peptide TAT (YGRKKRR) (Vives et al., 1997) at the N terminus to promote blood–brain barrier permeability [TAT-r3L4F (**3**); **Figure 1**] (Anastasio et al., 2013). A low dose of TAT-r3L4F (0.1 µmol/kg), which does not suppress motor activity on its own, enhances the efficacy of a subeffective dose of the selective 5-HT_2C_R agonist WAY163909 to suppress motor activity and impulsive action (Cunningham et al., 2011; Anastasio et al., 2013). Lastly, TAT-r3L4F blocks ∆^9^-tetrahydrocannabinol-induced conditioned place preference, an effect mimicked by the 5-HT_2C_R agonist Ro-600175 and reversed by 5-HT_2C_R antagonist SB242084 (Ji et al., 2006). Together, these results suggest that disruption of the 5-HT_2C_R-PTEN complex enhances 5-HT_2C_R-mediated effects *in vivo*.

In the present study, we first further investigated the effects of TAT-r3L4F *in vivo* with the use of a drug discrimination paradigm. Drug discrimination is a widely used rodent model for the assessment of the mechanism of action of novel compounds and has face validity as an assessment for the subjective effects of compounds in animals and humans (Appel and Cunningham, 1986; Schuster and Johanson, 1988; Teuns et al., 2014). Additionally, the abuse liability and similarity to an abused drug can be assessed using the drug discrimination model (Bubar and Cunningham, 2008). Notably, selective 5-HT_2C_R agonists suppress the stimulus effects of cocaine (Callahan and Cunningham, 1995) as well as other addiction-related behaviors (Bubar and Cunningham, 2008; Howell and Cunningham, 2015). Thus, in the present behavioral analyses, we explored the ability of TAT-r3L4F to affect the stimulus properties of selective 5-HT_2C_R agonists or cocaine. Next, our chemistry efforts focused on the design and synthesis of novel constrained peptide derivatives based on the sequence of peptide **2**. An alanine scan was employed to determine which amino acid residues were critical for *in vitro* peptide activity. Based on those results, cyclized peptides and peptidomimetic derivatives were designed and synthesized and shown to retain *in vitro* potency and efficacy.

## Materials and Methods

### Drug Discrimination Assays

#### Animals

Experimentally naïve male Sprague-Dawley rats (n = 43; Envigo, Inc.) weighing 300–325 g at the beginning of the experiment were housed two per cage in a temperature- (21–23°C) and humidity- (45–50%) controlled environment; lighting was maintained under a 12-h light–dark cycle (0700–1900 h). Rats were maintained at 80–90% of their free-feeding weights by restricting access to water. Rats received water during daily training sessions (5–6 ml/rat/session), in the afternoon several hours after training (20 min), and over the weekend (36 h). Experiments were conducted during the light phase of the light–dark cycle (between 0900 and 1200 h) and were carried out in accordance with the National Institutes of Health *Guide for the Care and Use of Laboratory Animals* and with the approval of the Institutional Animal Care and Use Committee at University of Texas Medical Branch.

#### Apparatus

The procedures were conducted in commercially available two-lever operant chambers (Med Associates, St. Albans, VT). Each chamber was equipped with a water-filled dispenser mounted equidistantly between two retractable response levers on the wall and housed in a light- and soundproof cubicle. Illumination came from a 28-V house light; ventilation and masking noise were provided by a ventilation fan in the right-side wall. A computer with Med-PC IV software was used to run programs and record all experimental events.

#### Drug Discrimination Procedure

Standard two-lever, water-reinforced drug discrimination procedures were used (Appel and Cunningham, 1986; Callahan and Cunningham, 1995; Colpaert, 1999). Three cohorts (n = 13–16/cohort) were trained to discriminate an injection of a training drug from saline (1.0 ml/kg, i.p.) administered 15 min before start of daily (Monday–Friday) training sessions. In one cohort (n = 14), rats were trained to discriminate the training drug lorcaserin (0.75 mg/kg, 1.0 ml/kg, i.p.) from saline. In a second cohort (n = 13), rats were trained to discriminate the training drug WAY163909 (0.75 mg/kg, 1.0 ml/kg, i.p.) from saline. In the third cohort, rats (n = 16) were trained to discriminate the training drug cocaine (5 mg/kg, 1.0 ml/kg, i.p.) from saline. Two rats in the lorcaserin–saline cohort were excluded from the present study due to failure to maintain the discrimination. Rats in the WAY163909–saline cohort also participated in additional studies (Wild et al., 2018); the WAY163909 dose–response curve was reevaluated between studies; a washout period was allowed, and no carryover effects were observed.

##### Errorless Training

During this phase, only the stimulus-appropriate (drug or saline) lever was present. Training began under a fixed ratio 1 (FR 1) schedule of water reinforcement, and the FR requirement was incremented until all animals were responding reliably under an FR 20 schedule for each experimental condition. For half of the rats, left lever responses were reinforced after training drug administration, whereas right lever responses were reinforced after saline administration; conditions were reversed for the remaining animals. During this phase of training, drug and saline were administered irregularly with the restriction that neither condition prevailed for more than three consecutive sessions.

##### Discrimination Training

After responding stabilized, both levers were presented simultaneously during 15-min training sessions. The rats were required to respond on the stimulus-appropriate (correct) lever to obtain water reinforcement. There were no programmed consequences for responding on the incorrect lever. This phase of training continued until the performance of all rats attained criterion (defined as mean accuracies of at least 80% stimulus-appropriate responding for 10 consecutive sessions).

##### Test Protocols

Test sessions were initiated and conducted once or twice per week following attainment of criterion. Training sessions were run during the intervening days to maintain discrimination accuracy. Rats were required to maintain accuracies of at least 80% correct for saline and training drug maintenance sessions that immediately preceded a test. During test sessions, animals were placed in the chamber, and upon completion of 20 responses on either lever, a single reinforcer was delivered, and the houselights were turned off. The rat was removed from the chamber, returned to the colony, and allowed free access to water for 15 min beginning 2–3 h after the end of each test. Test sessions were terminated after 15 min if the rats did not complete 20 responses on either lever; only data from rats that accomplished the FR 20 during test sessions within 15 min were employed in data analysis.

Two pharmacological test manipulations were performed during test sessions. Doses for all drugs were based on our previous publications (Cunningham et al., 2011; Anastasio et al., 2013; Neelakantan et al., 2017; Wild et al., 2018). In *substitution tests*, rats were administered various doses of the training drugs (lorcaserin–saline: 0.125–1.0 mg/kg lorcaserin, i.p.; WAY163909–saline: 0.125–1.0 mg/kg WAY163909, i.p.; cocaine–saline: 0.313–5 mg/kg cocaine, i.p.), saline, or test compounds. In *combination tests*, rats were tested for lever selection following intraperitoneal administration of a fixed dose of a test compound, or compounds, prior to a dose of the training drug. In lorcaserin–saline-trained rats, TAT-r3L4F (1, 2 µmol/kg; i.p.) was given 30 min prior to testing, and WAY163909 (0.75 mg/kg; i.p.) and lorcaserin (0.5 or 0.75 mg/kg; i.p.) were given 15 min prior to testing. In WAY163909–saline-trained rats, TAT-r3L4F (1, 2 µmol/kg; i.p.), lorcaserin (1 mg/kg; i.p.), and WAY163909 (0.5 or 0.75 mg/kg; i.p.) were given 15 min prior to testing. In cocaine–saline-trained rats, TAT-r3L4F (1–2 µmol/kg; i.p.) or SB242084 was given 45 min prior to testing, lorcaserin (0.5 or 1 mg/kg; i.p.) was given 30 min prior to testing, and cocaine (2.5 mg/kg; i.p.) was given 15 min prior to testing. Full substitution was defined as ≥80% drug-appropriate responding and not statistically different from the training drug and partial substitution as ≥40% and <80% drug-appropriate responding.

#### Drugs

TAT-r3L4F (**3**; Ac-YGRKKRRPNPDQKPRRKKKEKR-NH_2_; pepMic Co., China), lorcaserin [(1*R*)-8-chloro-2,3,4,5-tetrahydro-1-methyl-1*H*-3 benzazepine; Hangzhou Trylead Chemical Technology Co., Ltd, Hangzhou, China], WAY163909 [(7b-*R*,10a-*R*)-1,2,3,4,8,9,10,10a-octahydro-7bH-cyclopenta[b][1,4] diazepino [6,7,1hi] indole; gift from Pfizer, Inc., New York, NY], and (-) cocaine (National Institute on Drug Abuse, Research Triangle Park, NC) were dissolved in 0.9% NaCl for *in vivo* studies.

#### Data Analysis

Accuracy was defined as the percentage of correct responses to total responses before the delivery of the first reinforcer. During test sessions, performance was expressed as the percentage of drug-lever responses to total responses upon completion of an FR 20 on either lever. Response rates (responses per minute) were also evaluated during training and test sessions as a measure of behavioral disruption. The response rate (responses per minute) was calculated as the total number of responses emitted before completion of the first FR 20 divided by the number of minutes taken to complete the first ratio. For substitution tests, Student’s *t*-test for repeated measures was used to compare the percentage of drug-lever responding and response rate during test sessions with the corresponding values for the previous drug maintenance session. Combination tests were analyzed by one-tailed Student’s *t*-test for repeated measures with Bonferroni correction for preplanned comparisons. All statistical analyses were conducted with an experiment-wise error rate of α = 0.05.

### *In Vitro* Screening

#### Cell Lines and Cell Culture

Chinese hamster ovary K1 (CHO-K1) cells stably transfected with human unedited 5-HT_2C_R (h5-HT_2C_R-CHO cells) were a generous gift of K. Berg and W. Clarke (University of Texas Health Science Center, San Antonio). Cells were grown at 37°C, 5% CO_2_ and 85% relative humidity environment in GlutaMax™-MEM medium (Invitrogen, Carlsbad CA) containing 5% fetal bovine serum (Atlanta Biologicals, Atlanta GA), and 100 µg/ml hygromycin (Mediatech, Manassas VA), and were passaged when they reached 80% confluence.

#### Ligands

Serotonin (5-HT; Acros Organics, ThermoFisher Scientific, Pittsburgh, PA) was dissolved in 1X Hank’s balanced salt solution (HBSS; Cellgro, Invitrogen). Peptides were dissolved in DMSO to a concentration of 10 mM stock solutions for functional assays. PTEN inhibitor SF1670 (Echelon Biosciences, Salt Lake City, UT) was dissolved in DMSO to a stock concentration of 5 mg/ml.

#### Intracellular Calcium Assay

The Ca*_i_*^2+^ release assay was performed according to our recent publications with minor modifications (Seitz et al., 2012; Anastasio et al., 2013; Chen et al., 2017). Briefly, 150 µl of cells from passage 6-16 were plated in black-walled flat clear bottom 96-well tissue culture plates at a density of 14,000–16,000 cells/well in serum-replete medium. Care was taken to ensure even plating of cells, including frequent agitation or trituration of cells in the source reservoir; plates were placed on a rotary shaker at low speed for 20 min. Approximately 24 h following plating, the medium was removed, and cells were fed with serum-free (SF) GlutaMax^™^-MEM medium supplemented with 1-µM putrescine (Sigma-Aldrich, St. Louis, MO), 10-mM progesterone (Sigma-Aldrich), and 1:100 ITS (1,000 mg/l human recombinant insulin, 550 mg/l human recombinant transferrin, 0.67 mg/l selenious acid; Corning Inc, Corning, NY) (SF + medium). Following a 3-h incubation, SF + medium was replaced with 40 μl HBSS without CaCl_2_ or MgCl_2_ (pH 7.4) plus 40 μl Calcium 4 dye solution (FLIPR No-wash kit, Molecular Devices, Sunnyvale, CA, part # R8142) supplemented with 2.5 mM water-soluble probenicid (Sigma) to inhibit extracellular transport of the dye. Plates were incubated for 60 min at 37°C followed by 15 min at RT in the dark. Calcium-induced fluorescence signal (λex = 485 nm, λem = 525 nm) was measured with a FlexStation 3 instrument (Molecular Devices). A baseline was established for each well during the initial segment of each run. Addition of 20 μl of 5× concentrated peptide derivatives or vehicle (HBSS) occurred at 17 s, and fluorescence was recorded every 5 s for 240 s to determine intrinsic agonist activity. Fifteen minutes later, following another 17 s baseline recording, 25 μl of 5× concentrated 5-HT was added, and fluorescence was again measured every 1.7 s for 240 s. Maximum peak heights and area under the curve (AUC) of the Ca*_i_* ^2+^ transient were determined by the SoftMax software (Pro 5.4.5) for each well.

After the final readings, cells were fixed in 2% paraformaldehyde (Sigma) overnight. Data from each well were normalized to total cell mass as determined with crystal violet staining, a value proportional to cell mass that can be used as an estimate of cell number (Seitz et al., 2012; Anastasio et al., 2013; Chen et al., 2017). After fixation, cells were rinsed with water and air dried, and 50 µl of filtered crystal violet solution (0.1% in water) was added for 10 min at RT, and the wells were rinsed again. Cell-adsorbed dye was extracted by the addition of 50 µl of 10% acetic acid (10 min, RT) and absorbance read at 590 nm. AUC of the Ca*_i_* ^2+^ transient was normalized to the crystal violet values for each well and then expressed as a percent of the maximum and minimum Ca*_i_* ^2+^ response. The AUC of Ca*_i_* ^2+^ transients was utilized, as this measure incorporates both information on the duration of the calcium signal as well as the maximum amount of ligand-evoked calcium release (King et al., 2015). The pEC_50_ and E_MAX_ values for Ca*_i_* ^2+^ assay were determined using three-parameter nonlinear regression analysis (GraphPad Prism 7.02) and calculated from at least four independent experiments, each conducted in technical quadruplicates. Raw relative fluorescence units were then normalized to maximum Ca*_i_* ^2+^ release induced by 5-HT (100%) and are presented as the mean ± SEM% of 5-HT. An unpaired Welch’s *t*-test was conducted to compare E_MAX_ of 5-HT in the presence and absence of peptide derivative (1 nM). All statistical analyses were conducted with an experiment-wise error rate of α = 0.05.

#### PTEN Lipid Phosphatase Activity Assay

Lipid phosphatase activity of PTEN was quantified using a PTEN activity ELISA kit (K-4700, Echelon Biosciences, Salt Lake City, UT) following the manufacturer’s protocol. Briefly, 1 ng/µl PTEN enzyme (E-3000, Echelon Bioscience) was incubated with PTEN inhibitor SF1670 (200 µM), **1**, **2**, and **2** derivatives (10 µM) for 15 min at 37°C. Then, PI(3,4,5)P_3_ substrate was added for a final concentration of 4 µM, and the reaction was incubated at 37°C for 2.25 h. The lipid phosphatase activity of PTEN was quantified by measuring the amount of PI(4,5)P_2_ by competitive ELISA in which final absorbance is inversely related to the amount of PI(4,5)P_2_. The absorbance was measured by FlexStation3 at 450 nm (Molecular Devices). Results are represented as fold change over PTEN + PI(3,4,5)P_3_ alone (mean ± SD) of two independent experiments run in technical triplicates and analyzed by one-way ANOVA with *a priori* comparison with PTEN + PI(3,4,5)P_3_ alone (Dunnett’s; GraphPad Prism 7.02). All statistical analyses were conducted with an experiment-wise error rate of α = 0.05.

### Chemistry

#### General

All starting material and reagents were purchased from Sigma-Aldrich, Acros, AstaTech, and Aapptec and, unless noted, were used without further purification. The azide and alkyne were purchased or prepared by the methods of Pedersen. Thin-layer chromatography (TLC) was performed on Silicycle glass-backed plates (extra hard layer, 0.25-mm thick, 60 Å, with F-254 indicator), and components were visualized by UV light (254 nm) and/or *p*-anisaldehyde, basic permanganate (KMnO_4_) solution, ninhydrin solution. Flash column chromatography was performed using Silicycle silica gel (particle size 40–63 µm, 230–400 mesh). NMR spectra were obtained using JEOL ECX-400 spectrometer (400 MHz for ^1^H NMR and 100 MHz for ^13^C NMR), JEOL ECA-500 spectrometer (500 MHz for ^1^H NMR and 125 MHz for ^13^C NMR), or JEOL ECX-600 spectrometer (600 MHz for ^1^H NMR and 150 MHz for ^13^C NMR). Chemical shifts were referenced to the residual chloroform-H peak at 7.26 ppm (^1^H) and 77 ppm (^13^C) in CDCl_3_ or to DMSO-H peak at 2.5 ppm (^1^H) and 39.5 ppm (^13^C) in DMSO-*d*_6_ or to D_2_O at 4.79 ppm (^1^H). Chemical shifts were reported in parts per million (ppm, δ). Multiplicity was indicated as s for singlet, d for doublet, t for triplet, q for quartet, m for multiplet, and br for broad resonance, and the coupling constants (*J*) were reported in Hertz. High-resolution mass spectra were recorded on an Agilent 6530 Accurate Mass Q-TOF LC-MS (high-resolution ESI) by the University of Texas at Austin, Mass Spectrometry Facility of Department of Chemistry and Biochemistry. Low-resolution mass spectra were recorded on Thermo Scientific liquid chromatography mass spectrometry (LC-MS with low-resolution ESI) in Gilbertson Laboratory.

#### Analytical and Preparative RP-HPLC

Analytical RP-HPLC was run on an HP1100 series instrument using A. Thermo Scientific BetaBasic column (C8, 100 × 4.6 mm, 5-μm particle size with a flow rate of 0.8 ml/min), B. Beckman Coulter column (C18, 250 × 4.6 mm, 5-μm particle size with a flow rate of 1.0 ml/min), or C. Grace Vydac column (C18, 250 × 4.6 mm, 5-μm particle size with a flow rate of 1.0 ml/min). The analyses were executed with the following solvent systems: 0.1% TFA in H_2_O (A) and MeCN containing 0.1% TFA (B). Detection was performed with a photodiode array detector at a wavelength of λ = 215 nm unless otherwise stated. Preparative purification was performed on a Gilson series instrument using a Grace Vydac protein and peptide C18 column (C18, 250 × 18 mm, 10-μm particle size). The analyses were executed with a flow rate of 8–12 ml/min and with the following solvent systems: H_2_O containing 0.1% TFA (A) and MeCN (B).

#### General Method for Solid-Phase Peptide Synthesis

Automated solid-phase peptide synthesis (SPPS) was performed at room temperature on Endeavor 90 III peptide synthesizer (AAPPTEC). All syntheses were executed using a standard Fmoc/*^t^*Bu strategy. The resin [Rink amide resin or 2-Cl Trityl chloride (2-ClTrT) resin] was swollen in the solvent used in the reaction for 20 min prior to reaction. The first amino acid was attached to 2-ClTrT resin using the standard protocol provided in AAPPTEC technical support information bulletin 1027. The first amino acid attachment on Rink amine resin was introduced by standard coupling conditions. Briefly, the coupling reactions were carried out (for both Rink amide resin and 2-CTC resin) in NMP using 2 equiv of N-Fmoc-protected amino acid, HOBt (2 equiv), and HBTU (2 equiv) in the presence of DIPEA (5 equiv). The activated amino acid was then added to the resin and the vessel shaken for 25 min with nitrogen bubbling for the first 1 min, followed by draining of the solvent. The coupling procedure was performed twice. N-terminal Fmoc deprotection was achieved using 20% piperidine in DMF (vol/vol). The resin was washed with methanol, DMF, MeCN, and CH_2_Cl_2_ after every coupling and deprotection step. Peptide cleavage and side-chain deprotection were carried out by agitating the crude peptide-loaded resin in TFA cocktail solution (95% TFA:2.5% TIPS:2.5% H_2_O) for 3 h at room temperature or at 0°C. The ingredients were varied depending on the peptide sequence. The crude material was precipitated in cold diethyl ether, centrifuged, and purified by HPLC using 0–100% gradient in MeCN over 30 min. The purified aqueous solution was lyophilized to afford the final product. In the case of 2-ClTrT resin, peptide cleavage can be accomplished using AcOH/TFE/CH_2_Cl_2_ (1:1:8) solution to generate side-chain-protected peptide acid. The crude material was precipitated in 10–15 times the volume of hexane. The solvent was removed under reduced pressure to afford the side-chain-protected peptide, unless noted was used without further purification.

#### Cyclic peptide 4

H-Arg(bf)-2-CTC resin (500 mg, 0.29 mmole) was swollen in CH_2_Cl_2_ for 20 min prior to sythesis. The attachment of the next seven amino acid residues, Fmoc-Ala-OH, Fmoc-Asn(Trt)-OH, Fmoc-Gln(Trt)-OH, Fmoc-Asp(tBu)-OH, Fmoc-Gln(Trt)-OH, Fmoc-Asn(Trt)-OH, Fmoc-Pro-OH, and linker (Fmoc-GABA-OH), was conducted in an automated SPPS in which the iterative cycle (removal of the Fmoc group, wash, coupling of the next building block, wash) was performed consistently. The peptide-loaded-2-ClTrT resin was treated with 5 ml of AcOH/TFE/CH_2_Cl_2_ (1:1:8) solution for 30 min, filtered and washed the resin with 5 ml of 10% AcOH solution. 150 ml of hexane was added to the filtrate. Solvent was removed with a rotary evaporator (if the AcOH was not completely removed, more hexane can be added and continued evaporating the solvent until all the AcOH was removed) to give of crude peptide (591 mg, 0.26 mmol), which was dissolved in 3 ml of CH_2_Cl_2_, and DBU (79 mg, 0.52 mmol) was added. The reaction mixture was stirred for 30 min, and solvent was removed by rotary evaporator. The residue was purified by flash chromatography (using eluent gradient EtOAc to 1:9 methanol–dichloromethane) to afford peptide with desired free amine and carboxylic acid. Peptide (525 mg, 0.23 mmol), HBTU (95 mg, 0.25 mmol), and HOBt (38 mg, 0.25 mmol) were dissolved in 32 ml of DMF, and 10 min later, DIPEA (119 mg, 0.68 mmol) was added. The resulting mixture was stirred overnight at room temperature. The reaction mixture was quenched by the addition of 320 ml of H_2_O and was extracted with EtOAc (3 × 120 ml). The combined organic extracts were washed with 5% aqueous HCl (100 ml), sat. aqueous NaCl (100 ml), and H_2_O (100 ml), dried over MgSO_4_, filtered, and concentrated with rotary evaporator. The crude material was used without further purification. The crude material (500 mg) was treated with 20 ml of 95% TFA solution and stirred for 3 h at room temperature. The resulting solution was added to cold ether (400 ml), and the white precipitation was centrifuged, collected, dried under vacuum, and purified by RP-HPLC to give pure cyclic derivative **4** (38 mg) in 13% yield based on the initial resin loading. The NMR spectra were reported as a mixture of two conformations. ^1^H NMR (500 MHz, D_2_O) δ 4.70 (dd, *J* = 15.8, 9.2 Hz, 0.2H), 4.67–4.55 (m, 2.7H), 4.53 (dd, *J* = 8.7, 2.7 Hz, 0.2H), 4.37–4.28 (m, 2H), 4.28–4.15 (m, 2.7H), 3.69–3.51 (m, 1.8H), 3.47 (dd, *J* = 17.8, 9.8 Hz, 0.2H), 3.30–3.09 (m, 4H), 2.96 (dd, *J* = 17.1, 5.7 Hz, 1H), 2.93–2.84 (m, 3H), 2.84–2.71 (m, 2H), 2.52–2.38 (m, 1.8H), 2.38–2.21 (m, 5.4H), 2.21–2.07 (m, 2.2H), 2.07–1.82 (m, 6.2H), 1.82–1.68 (m, 3H), 1.68–1.50 (m, 2H), 1.43–1.36 (m, 3H). ^13^C NMR (126 MHz, D_2_O) δ 177.82, 177.79, 176.04, 174.88, 174.77, 174.63, 174.53, 174.33, 174.10, 173.34, 173.12, 173.04, 172.59, 172.33, 171.89, 156.73, 131.47, 60.82, 53.95, 53.62, 53.41, 50.81, 50.73, 50.39, 47.99, 40.45, 38.86, 35.92, 35.50, 35.08, 31.71, 31.27, 31.13, 30.91, 29.59, 27.81, 27.51, 26.21, 25.92, 24.57, 24.43, 23.79, 22.38, 16.77, 16.48.

#### Cyclic peptide 5

Bromoacetic acid anhydride (3.25 mmol), which was freshly generated by treating bromoacetic acid (903 mg, 6.50 mmol) with DIC (410 mg, 3.25 mmol) in DMF (13 mL), was added to the Rink amide resin (pre-generated free amine form, 1,030 mg, 0.65 mmol, 0.63 mmol/g), and the reaction vessel was shaken for 80 min, followed by standard wash. DMF (13 ml) was added to the resulting resin, followed by the addition of propargylamine, and allowed it to shake for 20 h at room temperature. The first amino acid, arginine, was attached to the alkyl-installed resin in a pre-generated anhydride (3.25 mmol) style. After the deprotection of Fmoc, the following seven amino acids, Fmoc-Ala-OH, Fmoc-Asn(Trt)-OH, Fmoc-Gln(Trt)-OH, Fmoc-Asp(tBu)-OH, Fmoc-Gln(Trt)-OH, Fmoc-Asn(Trt)-OH, Fmoc-Pro-OH, and linker (4-azidobutyric acid), were attached by SPPS. A plastic vessel was charged with the peptide-loaded resin, and 58 ml of DMSO (solvent was freshly deoxygenated by bubbling with nitrogen for 5 min), Cu(I)I (56 mg, 0.29 mmol), and 2,6-lutidine were added, and the vessel was sealed and shaken for 48 h. After the reaction, the resin was then washed with DMF, MeCN, H_2_O, sat. aqueous disodium EDTA solution (2 × 10 min), H_2_O, MeCN, CH_2_Cl_2_, and ether. The peptide was cleaved from resin with 95% TFA *via* standard protocol. The crude material was purified by RP-HPLC to give pure cyclic peptide **5** (298 mg) in 40% yield based on the initial resin loading. The NMR spectra were reported as a mixture of conformations. ^1^H NMR (600 MHz, D_2_O) δ 8.05 (s, 0.5H), 7.99 (s, 0.1H), 7.87 (s, 0.4H), 5.09 (d, *J* = 15.5 Hz, 0.3H), 5.03 (d, *J* = 15.3 Hz, 0.1H), 4.91 (m, 0.2H), 4.69 (m, 0.2H), 4.61 (ddd, *J* = 19.5, 12.5, 6.6 Hz, 2H), 4.57–4.51 (m, 1.3H), 4.50–4.34 (m, 2.6H), 4.33–4.28 (m, 0.5H), 4.25 (ddd, *J* = 16.4, 10.7, 6.3 Hz, 2H), 4.19–4.01 (m, 3.2H), 3.93 (d, *J* = 16.8 Hz, 0.1H), 3.59–3.36 (m, 2H), 3.12 (ddd, *J* = 20.6, 14.3, 6.9 Hz, 2H), 2.92 (dd, *J* = 17.1, 6.1 Hz, 0.9H), 2.86–2.76 (m, 2.7H), 2.75–2.69 (m, 2H), 2.50–2.41 (m, 0.5H), 2.41–2.16 (m, 6H), 2.16–2.02 (m, 4.2H), 2.02–1.83 (m, 5H), 1.76 (d, *J* = 6.8 Hz, 1H), 1.69 (dt, *J* = 14.3, 9.4 Hz, 1.6H), 1.59–1.51 (m, 1.8H), 1.48–1.34 (m, 0.7H), 1.25 (d, *J* = 7.2 Hz, 1.2H), 1.22 (d, *J* = 7.1 Hz, 1.5H), 1.19 (d, *J* = 7.2 Hz, 0.3H). ^13^C NMR (151 MHz, D_2_O) δ 177.66, 177.57, 177.53, 174.68, 174.62, 174.55, 174.43, 174.28, 174.17, 174.08, 173.97, 173.93, 173.74, 173.66, 173.51, 173.20, 173.01, 172.88, 172.55, 172.47, 172.32, 171.81, 156.67, 156.60, 143.04, 142.90, 60.73, 54.30, 54.09, 53.43, 51.11, 50.89, 50.51, 49.96, 49.70, 49.58, 49.49, 49.01, 47.90, 44.50, 40.55, 40.38, 36.16, 35.69, 35.21, 31.41, 30.98, 30.90, 30.49, 28.09, 26.02, 25.08, 24.80, 24.29, 16.59.

#### Side chain-to-side chain cyclic peptide 6

A Rink amide resin (300 mg, 0.189 mmol, 0.63 mmol/g)-bound desired peptide sequence was obtained by standard SPPS. The peptide-loaded resin was swollen in CH_2_Cl_2_ for 10 min. After the resin was filtered, a solution of Cu(I)Br (27 mg, 0.189 mmol) in 9 ml of DMSO (freshly deoxygenated by bubbling with nitrogen for 5 min at least), a solution of sodium ascorbate (37 mg, 0.189 mmol) in H_2_O (1.6 mL), 2,6-lutidine (203 mg, 1.89 mmol), and DIPEA (244 mg, 1.89 mmol), were added to resin in a plastic vessel. The mixture was purged with N_2_ for 5 min, and the vessel was sealed and shaken for 20 h at room temperature. The solvent was drained, and the resin was washed with DMF, MeCN, H_2_O, sat. aqueous disodium EDTA solution (2 × 10 min), H_2_O, MeCN, CH_2_Cl_2_, and ether. The resin was treated with 20% piperidine in DMF (2 × 10 ml) to deprotect the N-Fmoc. Finally, the peptide was cleaved from resin with 10 ml of TFA/TIPS/H_2_O (95:2.5:2.5) solution *via* standard protocol. The crude material was purified by RP-HPLC to give pure cyclic peptide **6** (40 mg) in 21% yield based on the initial resin loading. ^1^H NMR (600 MHz, DMSO-*d*_6_) δ 9.22 (br, 1H), 8.75 (d, *J* = 7.4 Hz, 1H), 8.55 (br, *J* = 5.3 Hz, 1H), 8.22 (d, *J* = 7.8 Hz, 1H), 8.15–8.07 (m, 2H), 8.05 (dd, *J* = 7.3, 3.7 Hz, 2H), 7.95 (s, 1H), 7.84 (d, *J* = 8.0 Hz, 1H), 7.54 (br, 1H), 7.43 (s, 1H), 7.33 (s, 1H), 7.27 (d, *J* = 18.9 Hz, 2H), 7.13 (s, 1H), 6.99 (s, 1H), 6.80 (d, *J* = 18.5 Hz, 2H), 4.63 – 4.56 (m, 2H), 4.53 (dd, *J* = 12.9, 6.1 Hz, 1H), 4.48 (d, *J* = 12.4 Hz, 1H), 4.43–4.35 (m, 2H), 4.32–4.20 (m, 4H), 4.15 (dd, *J* = 13.6, 7.8 Hz, 2H), 3.51 (dd, *J* = 9.5, 5.7 Hz, 1H), 3.26–3.20 (m, 1H), 3.20–3.15 (m, 1H), 3.08 (dd, *J* = 12.9, 6.6 Hz, 2H), 2.59 (dd, *J* = 15.8, 4.7 Hz, 1H), 2.44 (dd, *J* = 15.8, 8.9 Hz, 1H), 2.28 (dd, *J* = 8.1, 5.5 Hz, 1H), 2.13–2.07 (m, 2H), 2.00 (t, *J* = 8.0 Hz, 2H), 1.94–1.82 (m, 5H), 1.79–1.63 (m, 6H), 1.55–1.36 (m, 4H), 1.22 (d, *J* = 7.2 Hz, 3H), 1.04 (d, *J* = 7.5 Hz, 2H). ^13^C NMR (151 MHz, DMSO-*d*_6_) δ 174.03, 173.97, 173.14, 172.07, 171.38, 171.05, 170.94, 170.75, 170.41, 169.32, 168.10, 156.64, 143.22, 123.99, 68.86, 62.91, 58.87, 52.11, 52.07, 51.92, 51.84, 51.66, 50.16, 49.43, 48.44, 45.81, 40.39, 40.03, 36.80, 31.82, 31.35, 29.51, 29.26, 28.86, 28.47, 28.15, 25.01, 23.45, 22.28, 17.84.

Fmoc-Asn(Trt)-Pro-O*^t^*Bu (**28**). Fmoc-Asn(Trt)-OH (3,000 mg, 5.03 mmol), HBTU (2,098 mg, 5.53 mmol), and HOBt (847 mg, 5.53 mmol) were dissolved in DMF (25 ml), and DIPEA (1,946 mg, 15.08 mmol) was added. Ten minutes later, H-Pro-O*^t^*Bu (861 mg, 5.03 mmol) was added, and the mixture was allowed to stir overnight at room temperature. The reaction mixture was quenched by the addition of H_2_O (250 ml) and was extracted with EtOAc (3 × 100 ml). The combined organic extracts were washed with 5% aqueous HCl (100 ml), sat. aqueous NaCl (100 ml), and H_2_O (100 ml), dried over MgSO_4_, filtered, and concentrated with rotary evaporator. The residue was purified by silica gel flash chromatography (3:7 EtOAc-hexanes) to give **N**-Fmoc-protected dipeptide **28** (3,450 mg, 92%). The NMR spectra are reported for a mixture of two rotamers. ^1^H NMR (500 MHz, CDCl_3_) δ 7.75–7.61 (m, 2H), 7.53 – 7.38 (m, 2H), 7.27 (dt, *J* = 14.3, 6.9 Hz, 2H), 7.21–7.04 (m, 18H), 4.80 (dd, *J* = 12.4, 7.8 Hz, 0.67H), 4.64 (dd, *J* = 19.4, 7.5 Hz, 0.33H), 4.18 (t, *J* = 8.1 Hz, 1H), 4.14–4.08 (m, 1H), 3.97 (t, *J* = 7.3 Hz, 1H), 3.88 (br, 1H), 3.68–3.63 (m, 0.67H), 3.55 (dd, *J* = 13.9, 8.1 Hz, 0.33H), 3.34 (d, *J* = 8.5 Hz, 1H), 2.89–2.79 (m, 0.67H), 2.70 (t, *J* = 12.0 Hz, 0.33H), 2.59 (dd, *J* = 14.6, 4.3 Hz, 0.67H), 2.43 (d, *J* = 13.2 Hz, 0.33H), 1.98–1.89 (m, 1H), 1.89–1.72 (m, 3H), 1.31 (s, 6H), 1.28 (s, 3H). ^13^C NMR (126 MHz, CDCl_3_) δ 170.59, 168.50, 144.47, 143.85, 143.73, 141.10, 128.79, 128.62, 127.84, 127.75, 127.53, 127.01, 126.77, 125.30, 125.21, 119.77, 81.36, 70.74, 67.07, 59.70, 50.22, 47.10, 47.00, 39.32, 28.92, 27.96, 27.76, 24.67.

Fmoc-Ala-Arg(Pbf)-O^t^Bu (**34**).The procedure used was identical with that used for preparation of **28**. Yield is 90%. ^1^H NMR (500 MHz, CDCl_3_) δ 7.68 (d, *J* = 7.3 Hz, 2H), 7.53 (t, *J* = 8.6 Hz, 2H), 7.31 (dd, *J* = 11.4, 7.3 Hz, 2H), 7.20 (t, *J* = 7.3 Hz, 2H), 6.24 (br, 3H), 4.42–4.28 (m, 2H), 4.23 (d, *J* = 6.2 Hz, 2H), 3.97 (dd, *J* = 111.1, 104.3 Hz, 1H), 3.14 (br, 2H), 2.86 (s, 4H), 2.78 (s, 2H), 2.73 (s, 3H), 2.52 (s, 3H), 2.45 (s, 3H), 2.01 (s, 3H), 1.87–1.75 (m, 1H), 1.74–1.60 (m, 1H), 1.60–1.45 (m, 2H), 1.38 (s, 6H), 1.37 (s, 9H). ^13^C NMR (126 MHz, CDCl_3_) δ 173.17, 170.83, 158.58, 156.37, 156.13, 143.66, 143.40, 140.93, 138.09, 132.06, 127.48, 126.91, 125.04, 124.50, 119.66, 117.33, 86.23, 82.12, 67.17, 52.57, 50.47, 46.65, 42.94, 40.39, 38.35, 36.38, 31.21, 28.75, 28.35, 27.65, 24.88, 19.11, 18.34, 17.76, 12.28.

#### Turn Mimic 7

(S)-1-((S)-4-amino-2-((3S,4S)-4-((S)-1-((S)-1-carboxy-4-guanidinobutylamino)-1-oxo-propan-2-ylcarbamoyl)pyrrolidine-3-carboxamido)-4-oxobutanoyl)pyrrolidine-2-carboxylic acid (turn mimic **7**). The peptide fragments were coupled on to **36** by standard peptide amidation. N-Troc protected turn mimic (trans-pyrrolidine-3,4-dicarboxamide) was prepared by the procedure of Boger (Whitby et al., 2011) and was used for the preparation of **7** by standard peptide coupling methods. To a solution of the N-Troc, protected turn mimic **37** (120 mg, 0.09 mmol) in 1.8 ml of AcOH/THF (1:2) was added zinc dust (114 mg, 1.74 mmol). The resulting suspension was stirred at room temperature for 8 h and was then filtered through celite to remove zinc. The filtrate was concentrated with a toluene azeotrope to afford crude **38** (93 mg), which was employed directly into the next reaction without further purification. Three milliliters of TFA/TIPS/H_2_O (95:2.5:2.5) solution was added to the crude product (93 mg), and the mixture was stirred at room temperature for 3 h. Cold ether (30 ml) was added to the reaction mixture, and the resulting white precipitation was centrifuged, collected, dried under vacuum, and purified by RP-HPLC to give pure turn mimic **7** (33 mg, 71%). ^1^H NMR (500 MHz, D_2_O) δ 4.96 (dd, *J* = 10.2, 4.2 Hz, 1H), 4.40 (dd, *J* = 8.7, 4.9 Hz, 1H), 4.34 (dd, *J* = 9.1, 5.1 Hz, 1H), 4.30–4.20 (m, 1H), 3.79 (dt, *J* = 10.3, 6.6 Hz, 1H), 3.68 (dt, *J* = 10.0, 6.5 Hz, 1H), 3.62–3.50 (m, 4H), 3.36–3.25 (m, 2H), 3.19 (t, *J* = 6.9 Hz, 2H), 2.80 (dd, *J* = 15.7, 4.2 Hz, 1H), 2.56 (dd, *J* = 15.8, 10.2 Hz, 1H), 2.37–2.24 (m, 1H), 2.11–1.95 (m, 3H), 1.95–1.84 (m, 1H), 1.81–1.69 (m, 1H), 1.63 (tt, *J* = 13.5, 6.9 Hz, 2H), 1.37 (d, *J* = 7.4 Hz, 3H). ^13^C NMR (101 MHz, DMSO-*d*_6_) δ 173.34, 173.28, 172.93, 170.48, 170.33, 170.21, 170.00, 156.88, 58.77, 51.95, 48.83, 48.57, 47.11, 46.88, 46.50, 46.11, 40.34, 35.85, 28.60, 28.04, 25.23, 24.56, 17.70.

## Results

### Substitution and Combination Tests in Lorcaserin–Saline and WAY163909–Saline Cohorts

**Figure 2:**
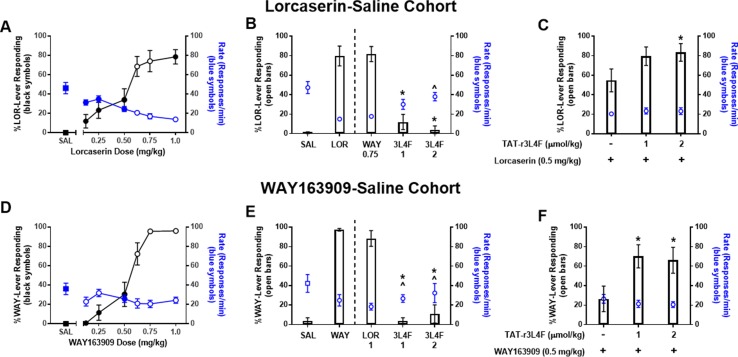
Results of dose–response tests in (top) rats (n = 14) trained to discriminate lorcaserin (LOR; 0.75 mg/kg) from saline and (bottom) rats (n = 7–13) trained to discriminate WAY163909 (WAY; 0.75 mg/kg) from saline (SAL). Black symbols denote the mean ( ± SEM) percentage of drug-lever responding; blue symbols denote the mean ( ± SEM) response rate per minute. For comparison, the percentage of drug-appropriate responding and response rate observed after saline tests are included (squares). Closed symbols indicate *p* < 0.05 vs. previous drug maintenance session. **(A-C)** Results of WAY163909 (WAY; 0.75 mg/kg), lorcaserin (LOR; 0.5-1 mg/kg), and TAT-r3L4F (3L4F; 1, 2 μmol/kg) substitution or combination tests in lorcaserin–saline-trained rats. *p < 0.05 vs. % lever response for LOR (0.5 mg/kg) alone. ^*p* < 0.05 vs rate of response for LOR (0.5 mg/kg) alone. For comparison, the percentage of drug-appropriate responding and response rate observed after training drug (0.75 mg/kg) and saline (SAL) are presented left of the dashed line. **(D-F)** Results of WAY163909 (WAY; 0.5-0.75 mg/kg), lorcaserin (LOR; 1 mg/kg), and TAT-r3L4F (3L4F; 1, 2 μmol/kg) substitution or combination tests in WAY163909–saline-trained rats. *p < 0.05 vs. previous % lever responses of drug maintenance session. ^*p* < 0.05 vs. previous rate of responses of drug maintenance session. Bars represent the mean percentage of drug (lorcaserin: LOR; WAY163909: WAY)-lever responding ( ± SEM) observed during test sessions. Blue symbols represent rate of response.

We first investigated the effects of the parent peptide TAT-r3L4F (**3**; **Figure 1**) with the use of a two-lever, water-reinforced drug discrimination paradigm in two separate cohorts of rats as a measure of intrinsic 5-HT_2C_R agonist activity *in vivo* (**Figure 2**). Note, the TAT-conjugated TAT-r3L4F peptide was employed in all rodent experiments. All rats in both the lorcaserin–saline and WAY163909–saline cohorts acquired the discrimination within similar number of training sessions. Rats acquired the discrimination of lorcaserin (0.75 mg/kg) versus saline within an average of 48 training sessions (range 33–71); response rates after lorcaserin were lower than rates after saline (*p* < 0.05). Rats acquired the discrimination of WAY163909 (0.75 mg/kg) versus saline within an average of 58 training sessions (range 39–107); response rates after WAY163909 were lower than rates after saline (*p* < 0.05). Substitution tests indicated that saline engendered <10% drug-lever responding in both cohorts (**Figure 2**). Both lorcaserin and WAY163909 evoked similar dose-related (0.125–1 mg/kg) increases in drug-appropriate responding as well as suppression of response rates (**Figure 2**).

In the lorcaserin–saline-trained rats, drug-appropriate lever responding after lorcaserin doses of 0.125, 0.25, 0.5, and 1 mg/kg were significantly different from the previous lorcaserin maintenance session (*p* < 0.05; **Figure 2A**). Doses of 0.125, 0.25, and 0.5 mg/kg produced response rates significantly elevated versus previous lorcaserin maintenance sessions (*p* < 0.05; **Figure 2A**). The dose of lorcaserin that predicted to elicit 50% lorcaserin-lever responding (ED_50_) was 0.54 mg/kg (95% CI 0.46–0.62 mg/kg). Given that lorcaserin and WAY163909 are similar in structure and have similar (but not identical) pharmacological properties (Dunlop et al., 2006; Thomsen et al., 2008), we performed cross-substitution analyses. WAY163909 (0.75 mg/kg) induced a full substitution in the lorcaserin–saline-trained cohort and suppressed response rates similar to the training drug (**Figure 2B**), as expected. Treatment with TAT-r3L4F (1, 2 µmol/kg) resulted in <10% drug-like responding in lorcaserin–saline-trained rats (*p* < 0.05; **Figure 2B**), suggesting that TAT-r3L4F alone does not induce interoceptive cues associated with this 5-HT_2C_R agonist. Treatment with TAT-r3L4F (2 µmol/kg) resulted in response rates that were significantly higher than the rate on the previous lorcaserin maintenance session in the lorcaserin–saline cohort (with a trend for 1 µmol/kg TAT-r3L4F) (*p* < 0.05; **Figure 2B**). TAT-r3L4F (2 µmol/kg) that did not substitute for lorcaserin, enhanced drug-lever responding when combined with lorcaserin (0.5 mg/kg; *p* < 0.05; **Figure 2C**) with no change in response rates.

In the WAY163909–saline-trained rats, drug-appropriate lever responding after WAY163909 (0.125, 0.25, 0.5 mg/kg) was significantly different from the previous WAY163909 maintenance session (*p* < 0.05; **Figure 2D**), while response rates were different at 0.5 mg/kg (*p* < 0.05; **Figure 2D**). The ED_50_ of WAY163909 was 0.56 mg/kg (95% CI 0.51–0.61 mg/kg). Additionally, lorcaserin (1 mg/kg) induced a full substitution in the WAY163909–saline-trained cohort and suppressed response rates versus saline (**Figure 2E**). Treatment with TAT-r3L4F (1, 2 µmol/kg) resulted in <10% drug-like responding in WAY163909–saline-trained rats (*p* < 0.05; **Figure 2E**), suggesting that TAT-r3L4F alone does not induce interoceptive cues associated with this 5-HT_2C_R agonist. Rates elicited by both tested doses of TAT-r3L4F were significantly higher than previous WAY163909 maintenance session in the WAY163909–saline cohort (*p* < 0.05; **Figure 2E**). The same doses of TAT-r3L4F (1, 2 µmol/kg) that did not substitute for WAY163909 versus saline discrimination enhanced drug-lever responding when combined with WAY163909 (0.5 mg/kg; *p* < 0.05; **Figure 2F**) with no change in response rates. These data suggest that disruption of the 5-HT_2C_R-PTEN complex by TAT-r3L4F (Anastasio et al., 2013) enhances the interoceptive cues elicited by lorcaserin or WAY163909.

### Substitution and Combination Tests in Cocaine-Saline Cohort

**Figure 3:**
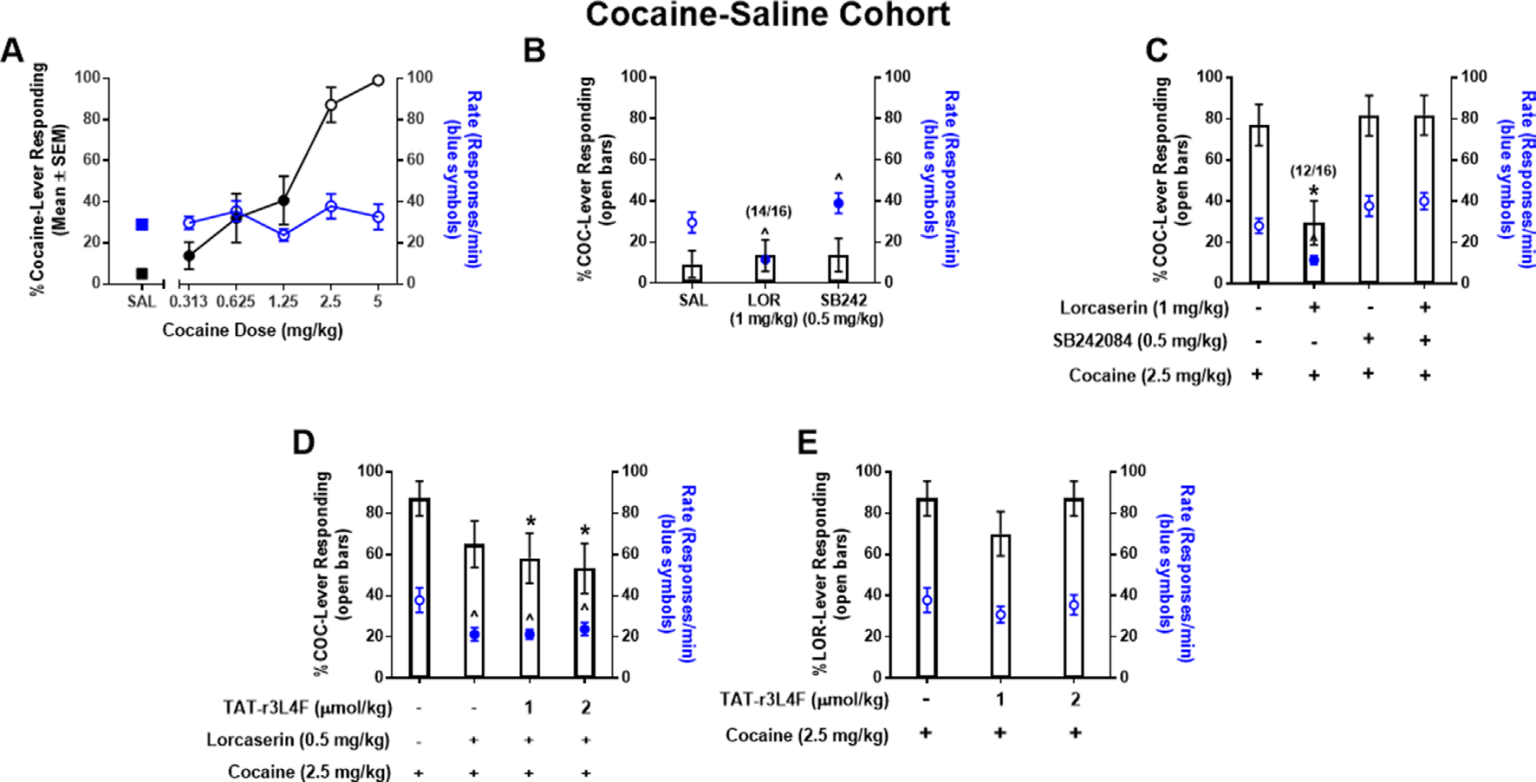
**(A)** Results of dose–response tests in rats (n = 16) trained to discriminate cocaine (COC; 5 mg/kg) from saline. Black symbols denote the mean (±SEM) percentage of cocaine-lever responding; blue symbols denote the mean (±SEM) response rate per minute. For comparison, the percentage of cocaine-appropriate responding and response rate observed after saline test are included (SAL: squares). Closed symbols indicate *p* < 0.05 vs. previous drug maintenance session. *n/N* indicates the number of rats completing the fixed ratio 20 versus the number of rats tested. Results of substitution tests **(B)** with saline (1 ml/kg), lorcaserin (1 mg/kg), and SB242084 (0.5 mg/kg) and combination tests **(C)** with cocaine (2.5 mg/kg), lorcaserin (1 mg/kg), and SB242084 (0.5 mg/kg). Bars represent the mean percentage of cocaine (COC)-lever responding (±SEM) observed during indicated test session. Blue symbols represent rate of response following tests. ^*p* < 0.05 vs. rate of response of previous cocaine maintenance session; *p < 0.05 vs. cocaine (2.5 mg/kg). **(D**, **E)** Results of combination tests with cocaine (2.5 mg/kg), lorcaserin (0.5 mg/kg), and TAT-r3L4F (1, 2 μmol/kg). Bars represent the mean percentage of cocaine (COC)-lever responding (±SEM) observed during indicated combination test session. Blue symbols represent rate of response following combination tests. *p < 0.05 vs. % lever responding of cocaine (2.5 mg/kg); ^p < 0.05 vs. Rate of responds of cocaine (2.5 mg/kg).

Cocaine produces robust subjective effects that contribute to its abuse (Kleven et al., 1990), and, as such, molecules that suppress the stimulus effects of cocaine may have important clinical implications. Here, we hypothesized that TAT-r3L4F would potentiate selective 5-HT_2C_R agonist-induced suppression of the stimulus effects of cocaine. To test this, we trained a third cohort of rats to discriminate cocaine (5 mg/kg) from saline. All rats acquired the discrimination of cocaine (5 mg/kg) versus saline within an average of 36 two-lever training sessions (range 32–42); response rates after cocaine (25.6 ± 0.55/min) were not statistically different from rates after saline (27.2 ± 1.3/min). During dose–response tests, cocaine (0.313–5 mg/kg) produced a dose-dependent increase in cocaine-appropriate responding (**Figure 3A**). Saline engendered <10% cocaine-lever responding (**Figure 3A**). The mean response rates after cocaine (0.313–5 mg/kg) did not differ significantly from those on the previous cocaine maintenance session. As expected (Callahan and Cunningham, 1995; Filip and Cunningham, 2002; Frankel and Cunningham, 2004), both lorcaserin (1 mg/kg) and selective 5-HT_2C_R antagonist SB242084 (0.5 mg/kg) engendered primarily saline-lever responding, indicating that neither drug substituted for cocaine (**Figure 3B**). Lorcaserin (1 mg/kg) produced a significant reduction in response rate versus previous cocaine maintenance session rates, while SB242084 (0.5 mg/kg) produced a slight but significant increase in response rate (*p* < 0.05; **Figure 3B**). Two rats failed to complete the FR 20 schedule after administration of lorcaserin (1 mg/kg), indicating that this dose induces behavioral disruption (**Figure 3B**). A full substitution of 2.5 mg/kg of cocaine was observed (**Figure 3C**). Pretreatment with lorcaserin (1 mg/kg) significantly reduced cocaine lever-appropriate responding (*p* < 0.05; **Figure 3C**) and response rates (*p* < 0.05; **Figure 3C**) consistent with previous reports that 5-HT_2C_R agonists suppress the stimulus effects of cocaine (Callahan and Cunningham, 1995; Filip and Cunningham, 2002; Frankel and Cunningham, 2004). Four rats failed to complete the FR 20 schedule after administration of lorcaserin (1 mg/kg) plus cocaine (2.5 mg/kg), indicating that this combination induces behavioral disruption (**Figure 3C**). Pretreatment with SB242084 did not alter cocaine lever-responding or response rate (**Figure 3C**). The lorcaserin-mediated suppression of cocaine on lever responding and response rate was fully recovered by SB242084 pretreatment (**Figure 3C**), further supporting that lorcaserin suppresses the stimulus effects of cocaine and response rate *via* 5-HT_2C_R agonism.

We then investigated whether TAT-r3L4F pretreatment would potentiate an ineffective dose (0.5 mg/kg) of lorcaserin to suppress the stimulus effects of cocaine. In substitution tests, TAT-r3L4F alone (1, 2 µmol/kg), lorcaserin alone (0.5 mg/kg), and the combination of TAT-r3L4F (1, 2 µmol/kg) plus lorcaserin (0.5 mg/kg) evoked saline-appropriate responding and significant reduction in response rates versus the previous cocaine maintenance session (data not shown). Cocaine (2.5 mg/kg) evoked full substitution that was not suppressed by lorcaserin (0.5 mg/kg) alone (**Figure 3D**), indicating that at this dose, lorcaserin does not suppress the stimulus effects of cocaine. Pretreatment with TAT-r3L4F (1, 2 µmol/kg) plus lorcaserin (0.5 mg/kg) significantly reduced cocaine-appropriate responding versus cocaine alone (*p* < 0.05; **Figure 3D**), suggesting that TAT-r3L4F enhances the efficacy of lorcaserin to suppress the stimulus effects of cocaine. However, the triple combination (TAT-r3L4F plus lorcaserin plus cocaine) is not statistically different from lorcaserin alone plus cocaine (**Figure 3D**). Both lorcaserin alone (0.5 mg/kg) and the combination of TAT-r3L4F (1, 2 µmol/kg) plus lorcaserin (0.5 mg/kg) significantly suppressed the response rates associated with cocaine (*p* < 0.05; **Figure 3D**). Treatment with TAT-r3L4F in the absence of lorcaserin did not alter the stimulus effects of cocaine or the associated response rate (**Figure 3E**).

### Alanine Scan

**Figure 4:**
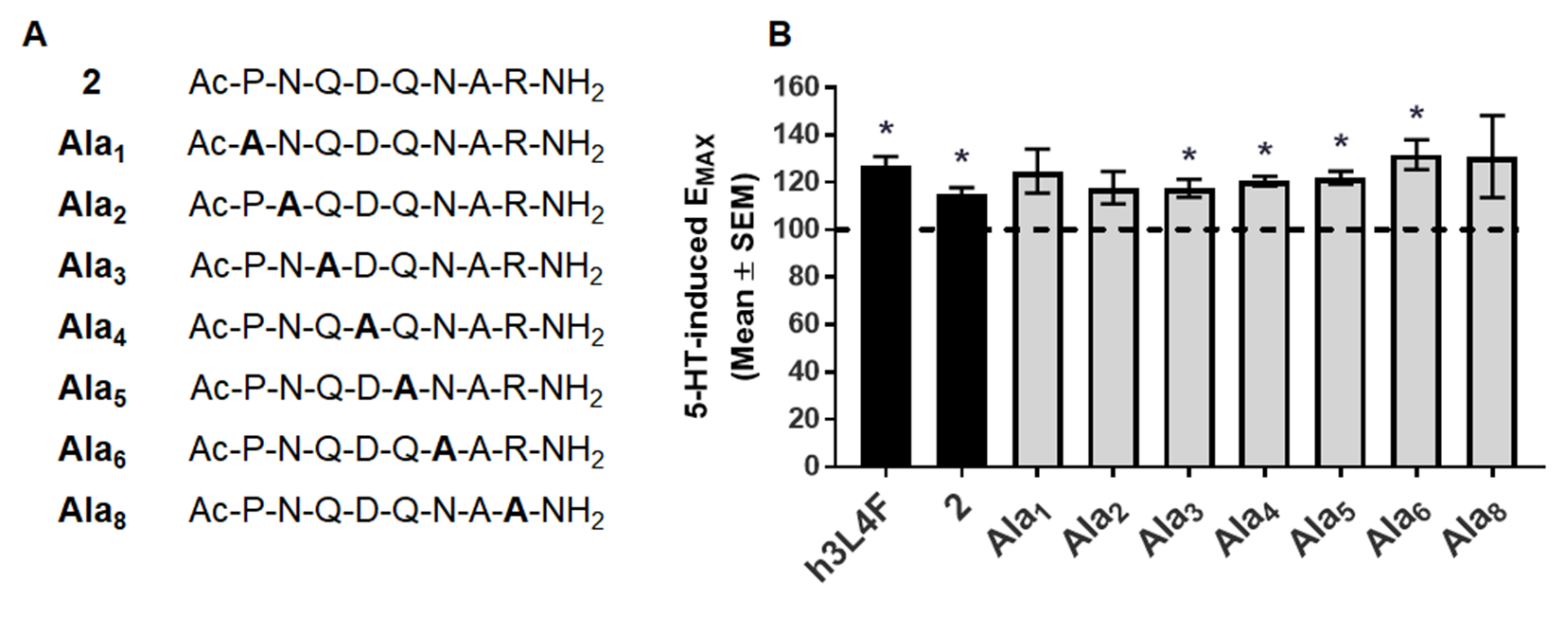
Alanine derivatives of peptide 2 **(A)** were **(B)** tested in Ca*_i_* ^2+^ release assay in live h5-HT_2C_R-CHO cells in the presence of 5-HT. The maximum 5-HT-induced Ca*_i_* ^2+^ release in the absence of the compounds was set as 100% (dashed line). Bars represent the average E_MAX_ produced by 1 nM alanine derivative in the presence of 5-HT, shown as mean ± SEM of 4–5 biological replicates. *p < 0.05 vs. 5-HT alone (dashed line).

Next, we continued to explore the 5-HT_2C_R-PTEN interaction *in vitro* by developing constrained peptide derivatives *via* cyclization of the peptide **2** sequence as well as replacement of peptide backbone with a rigid, non-peptide linker. We began with peptide **2** (**Figure 1**), an eight amino acid derivative of h3L4F, as the scaffold due to its retention of activity *in vitro* (Anastasio et al., 2013). The initial step in the generation of cyclized and peptidomimetic derivatives was to identify the amino acid residues of peptide **2** that could be modified without loss of *in vitro* activity. Each amino acid in the peptide **2** sequence was sequentially replaced with an alanine that resulted in seven alanine peptide analogs, since amino acid 7 is already an alanine (**Figure 4A**). Alanine replacement, i.e. “alanine scan”, was used to determine the amino acid side chains critical for the activity of the peptide (Morrison and Weiss, 2001). The ability of these alanine analogs to potentiate 5-HT-induced Ca*_i_* ^2+^ release was tested in h5-HT_2C_R-CHO cells. Serotonin induces a concentration-dependent increase in Ca*_i_* ^2+^ release (pEC_50_ = 8.1 ± 0.1, EC_50_ = 7.8 nM). Pretreatment with h3L4F or peptide **2** elevates the maximum Ca*_i_* ^2+^ release induced by 5-HT by ∼30% and ∼20%, respectively (*p* < 0.05; **Figure 4B**), with no change in EC_50_ versus 5-HT alone (data not shown). As shown in **Figure 4B**, analogs with an alanine replacement in positions 3–6 retained significant potentiation of 5-HT-induced Ca*_i_* ^2+^ release (*p* < 0.05; **Ala** **_3_**, E_MAX_ = 118 ± 3.8; **Ala** **_4_**, E_MAX_ = 121 ± 2.0, **Ala** **_5_**, E_MAX_ = 122 ± 2.7; **Ala** **_6_**, E_MAX_ = 132 ± 6.4) that is comparable with the parent peptides h3L4F (E_MAX_ = 131 ± 6.3) and **2** (E_MAX_ = 118 ± 4.0). This suggests that the Q-D-Q-N sequence in the middle portion of the peptide can be manipulated without loss of activity. Analogs **Ala** **_1_**(E_MAX_ = 125 ± 9.3), **Ala** **_2_** (E_MAX_ = 118 ± 6.8), and **Ala** **_8_** (E_MAX_ = 131 ± 17) did not significantly potentiate 5-HT-induced Ca*_i_* ^2+^ release (**Figure 4B**). Thus, amino acids in positions 1, 2, and 8 were not manipulated in subsequent modifications.

### Synthesis of Peptide Derivatives 4, 5, and 6

**Figure 5:**
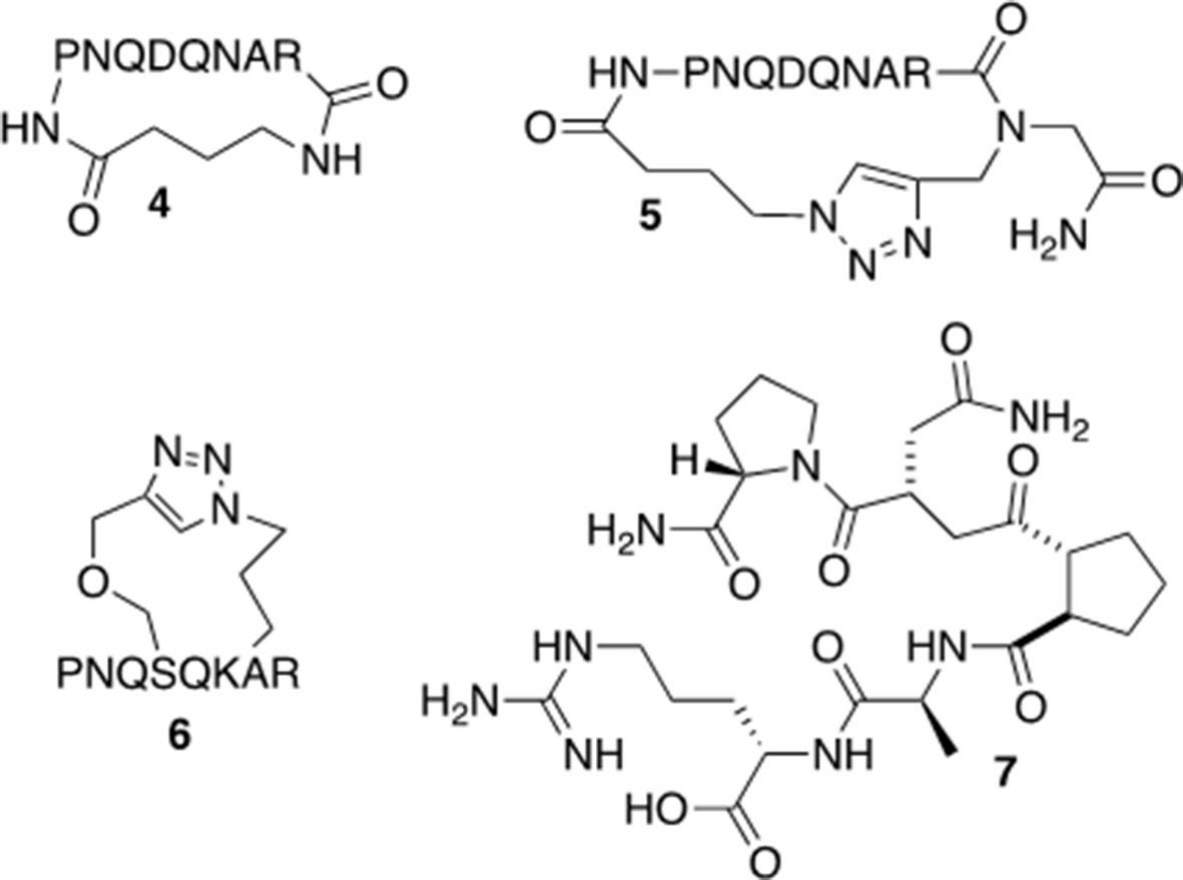
Modified versions of peptide 2.

Based on the results from the alanine scan and previous work in which modeling of peptide **2** indicates a potential preference for a turn-type conformation (Anastasio et al., 2013), peptide derivatives of **2** were synthesized (**Figure 5**). The head-to-tail cyclized versions (**4** and **5**) were designed to improve the stability of the molecules and limit their conformational flexibility (Venkatesan and Kim, 2002). Peptide derivative **6** was cyclized through the side chains in an *i, i+2* orientation in order to mimic a turn-type conformation. Peptide derivative **7** was designed to be a peptide turn-mimic in which the pyrrolidine-3,4-dicarboxamide replaced the middle four amino acids but retained the necessary orientation for the amino acids at the N and C terminal ends (see below) (Whitby et al., 2011). In addition to increasing *in vitro* stability, these constraints should limit the number of peptide conformations available and may affect the activity of the peptide, although the pharmacokinetic properties of these derivatives have not been tested.

**Scheme 1:**
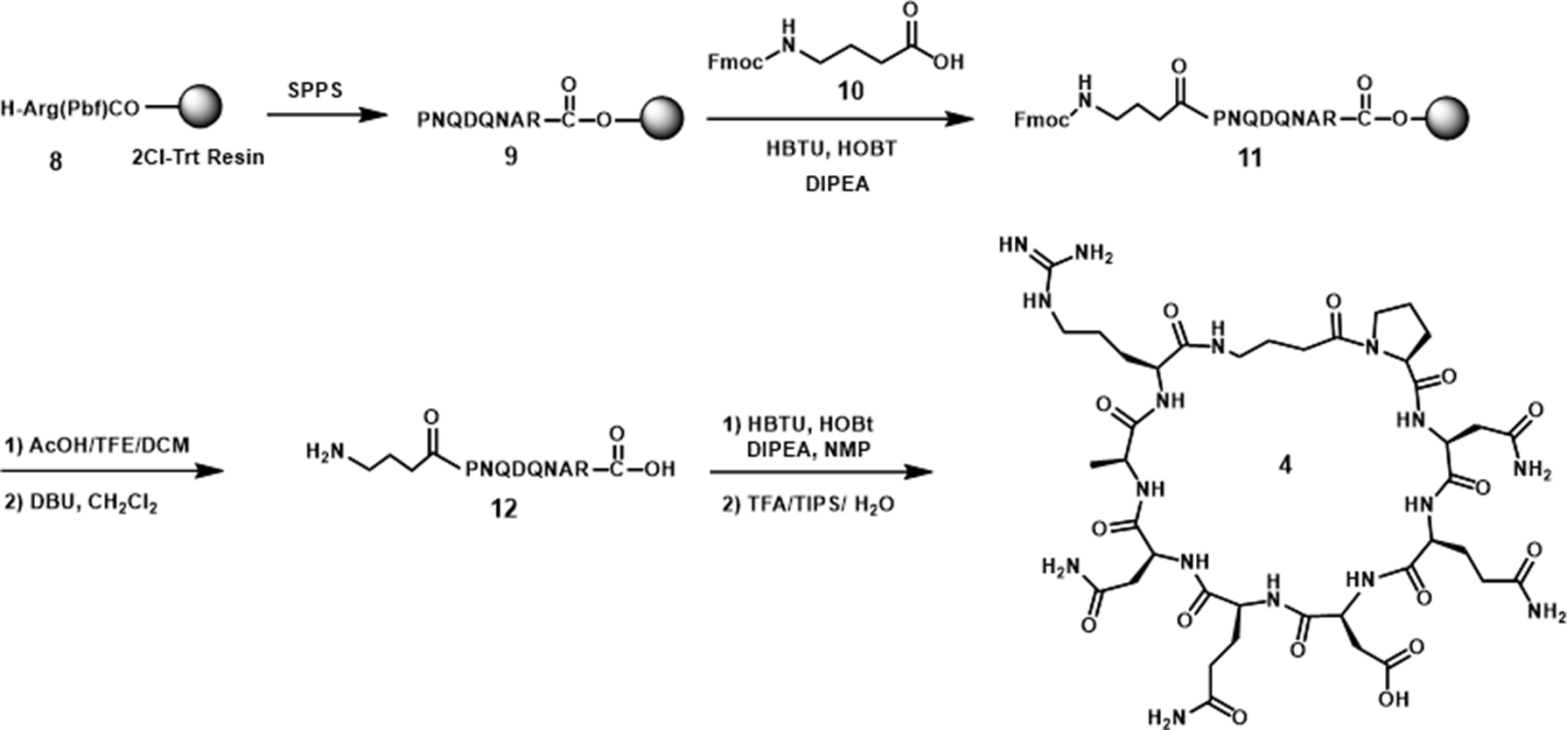
Synthesis of cyclic peptide **4**.

The amide cyclized peptide derivative **4** was synthesized starting with a linear precursor with side chains protected but a free amine and acid in the N- and C-terminus (**Scheme 1**). The linear peptide was synthesized by using an arginine preloaded 2-chlorotrityl chloride (2-CTC) resin, which can be cleaved under mild acid conditions without deprotecting the peptide side chains. After standard peptide coupling to provide the sequence, Fmoc-4-amino butyric acid (**10**) was added to the amino end of the linear peptide. Cleavage from the polymer resin was accomplished by treating with 10% of AcOH in CH_2_Cl_2_ followed by the removal of Fmoc from the amino butyric acid end in the presence of DBU in CH_2_Cl_2_. The key intramolecular macrocyclization was performed under high-dilution conditions using HBTU/HOBt as the coupling reagents and DIPEA as the base. The side-chain-protecting groups (Trt, ^t^Bu, and Pbf groups) were then removed in a single step with TFA solution (TFA/TIPS/H_2_O 95:2.5:2.5) to give cyclized peptide derivative **4**. The crude material was purified by reverse-phase HPLC to furnish pure cyclic peptide in 13% yield based on the initial resin loading.

**Scheme 2:**
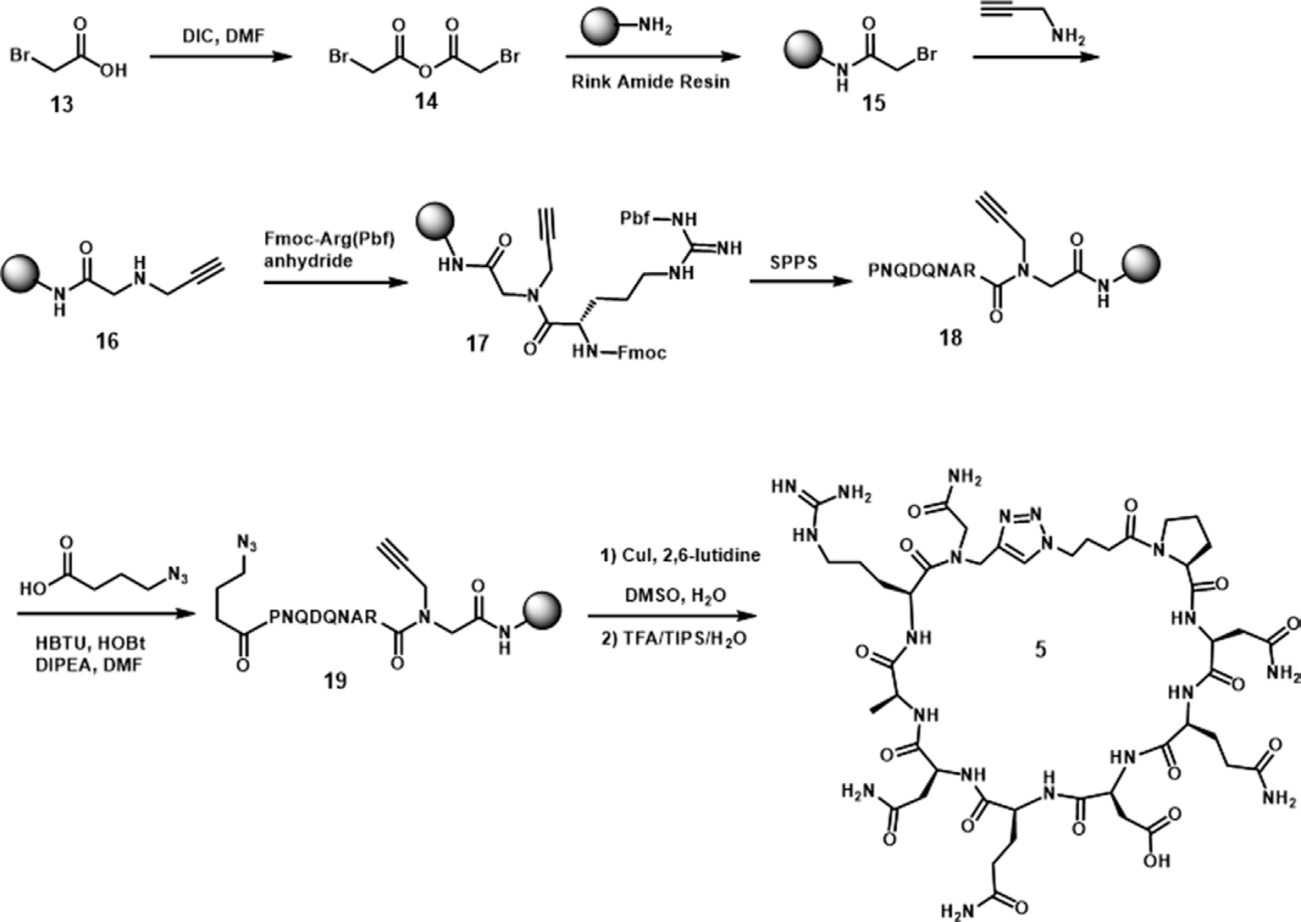
Synthesis of cyclic peptide **5**.

Standard amide formation and copper catalyzed azide-alkyne cycloaddition (CuAAC) were used to perform cyclizations in the approach to **5** and **6**. The cyclic peptide **5** was cyclized by formation of a 1,2,3-triazole ring (**Scheme 2**) (Jagasia et al., 2009; Ingale and Dawson, 2011). The synthesis was started with the reaction of bromoacetic anhydride with Rink amide resin to afford **15**. The bromide was then substituted by propargyl amine to incorporate the necessary alkyne for triazole ring formation (**16**). The first amino acid, Arg, was coupled to the resultant secondary amine by using a presynthesized symmetrical anhydride of arginine. After the incorporation of the necessary amino acids by solid-phase peptide synthesis, the azide was introduced by the attachment of 4-azidobutyric acid. The cyclization was carried out through an on-resin strategy in the presence of Cu(I) catalyst and 2,6-lutidine in DMSO. The cyclic peptide was then cleaved from the resin to give **5** in 40% yield, based on the initial loading of the resin.

**Scheme 3:**
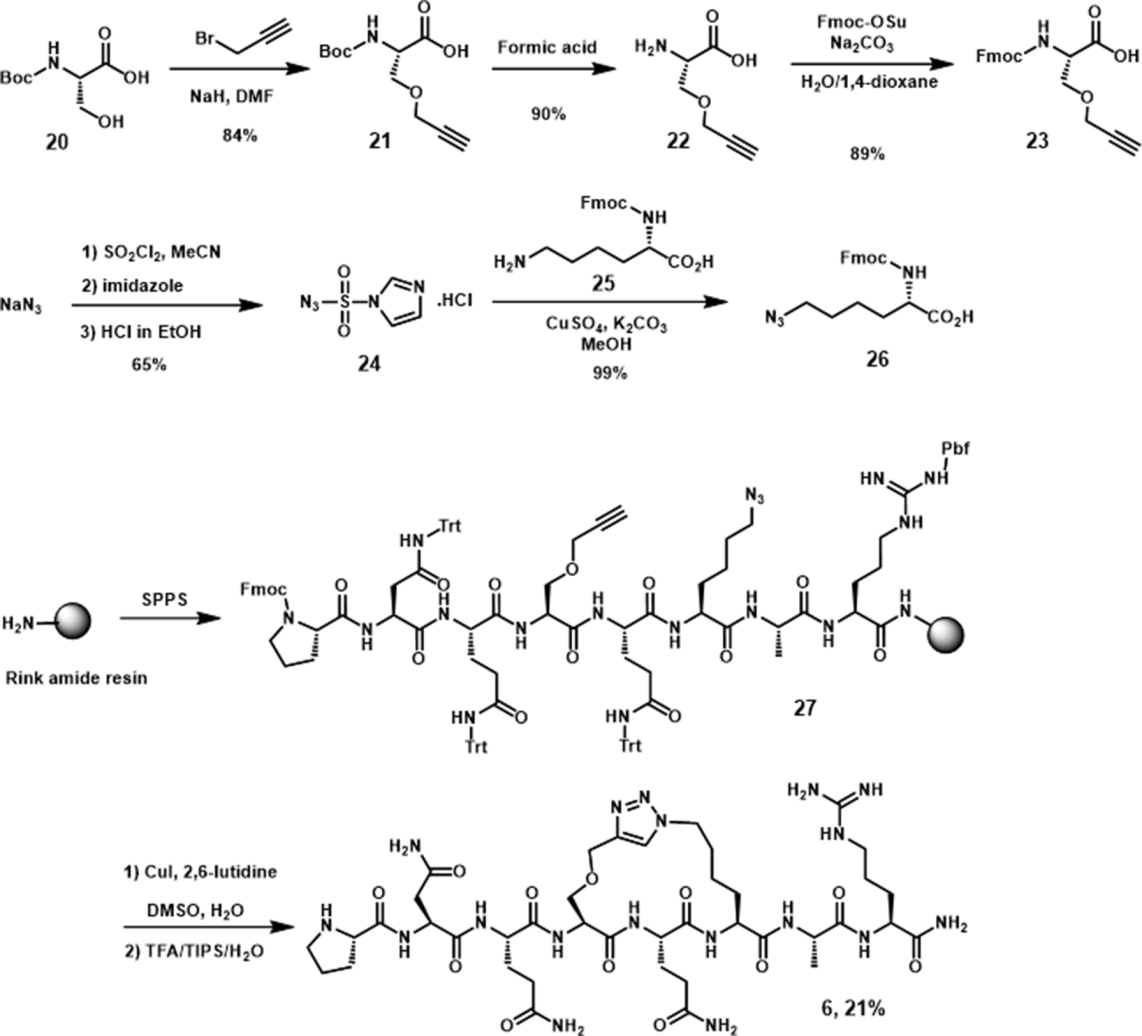
Synthesis of *N*-Fmoc peptides **23**, **26**, and peptidomimetic **6**.

The synthesis of **6**, the peptide derivative linked through side chains in *i, i+2* positions, was performed by using a similar procedure as utilized for **5**, with two presynthesized alkyne and azide moieties (**Scheme 3**) incorporated in the peptide sequence. After linear precursor **27** was generated, the cyclic peptide derivative **6** was obtained on the resin by Cu(I) catalyzed cycloaddition followed by subsequent TFA deprotection and cleavage.

### Synthesis of Turn Mimic 7

**Scheme 4:**
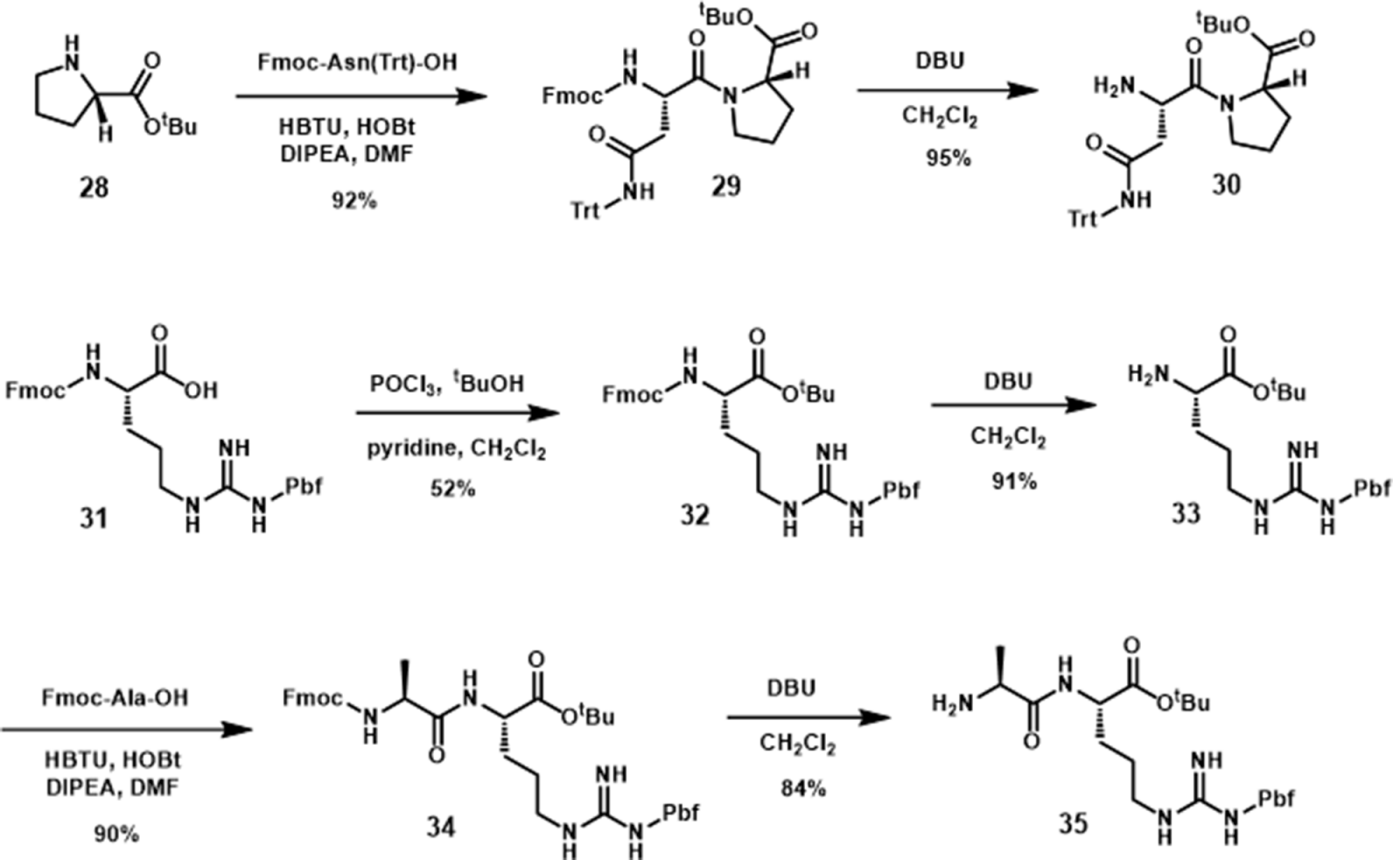
Synthesis of dipeptides **30** and **35**.

**Scheme 5:**
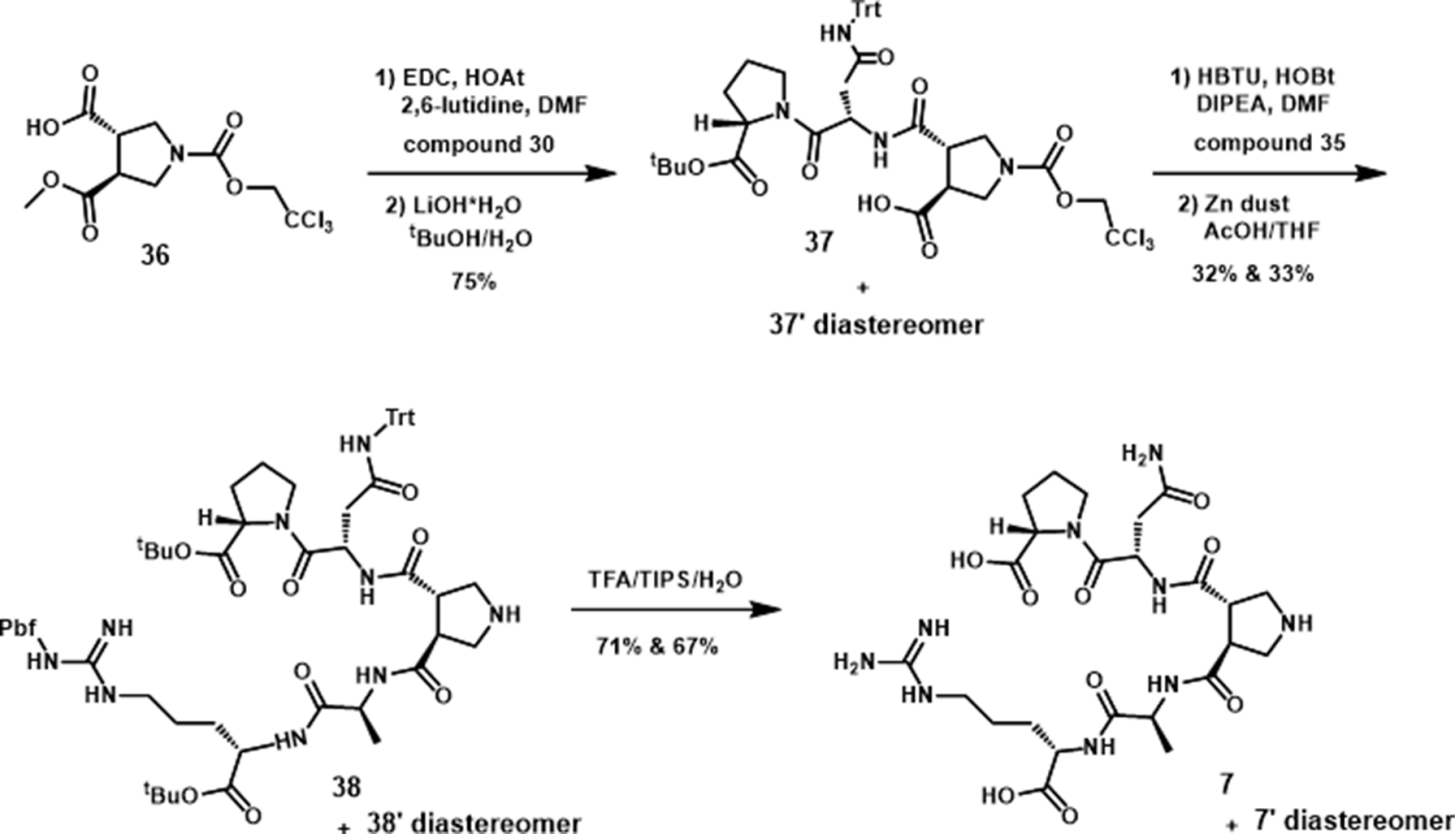
Synthesis of diastereomeric β-turn peptidomimetics **7** and **7**’.

The synthesis of the pyrrolidine-3,4-dicarboxamide was carried out according to the report of Boger (Whitby et al., 2011). With this β-turn template **36** in hand, we moved our synthetic efforts to the preparation of peptide fragments, which were installed on the template. In order to achieve the maximum similarity of the 3L4F sequence, two amino acid residues of each (N- and C-) terminus were preserved in the synthesis (**Scheme 4**). The first fragment was synthesized by coupling the proline *tert*-butyl ester amine with Fmoc-Asn(Trt)-OH, followed by removal of Fmoc to yield amine **30** in 87% yield. The fragment for the other position on the pyrrolidine was obtained by esterification of commercial Fmoc-Arg(Pbf)-OH (**31**) with ^t^BuOH in the presence of POCl_3_ and pyridine (52% yield) followed by deprotection of N-terminal Fmoc with DBU (91% yield). Amide formation with alanine and deprotection provided dipeptide **35** in 76% yield (**Scheme 4**). Dipeptide **30** was coupled to **36** (**Scheme 5**) by reaction with HOAt, and the subsequent acid hydrolysis produced the diastereomers **37** and **37’** (75% yield). After addition of dipeptide **35** and separation of the diastereomers, the removal of Troc group was conducted *via* EDC/HOAt and zinc dust to give **38** and **38’** in 32% and 33% yield, respectively. A solution of TFA cocktail solution was used to remove all other protecting groups in one step to give the crude material by cold ether precipitation. Finally, the diastereomeric β-turn peptidomimetics **7** and **7’** were obtained (71% and 67% yields) after the preparative HPLC system purification.

### *In Vitro* Assessment of Peptide Derivatives

**Figure 6:**
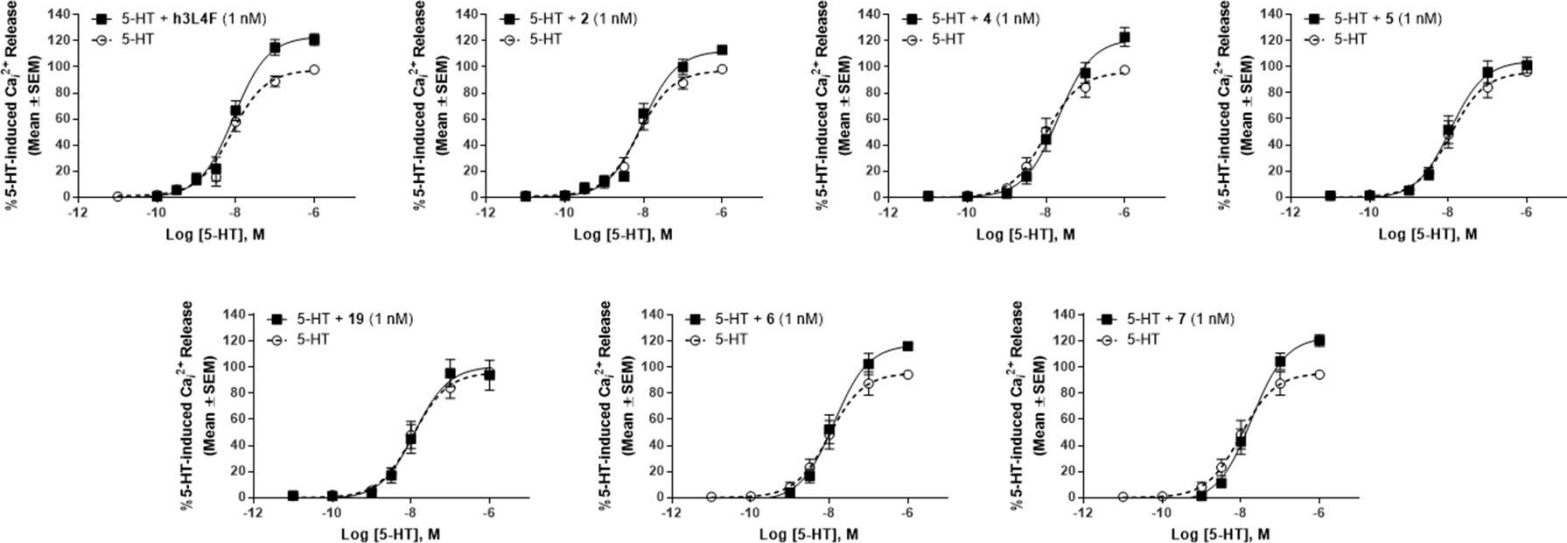
Effects of peptide **2** derivatives on Ca*_i_* ^2+^ release in live h5-HT_2C_R-CHO cells in the absence (open circles) and presence of 1 nM compound (closed circles) against the concentration–response curve for 5-HT. The maximum 5-HT-induced Ca*_i_* ^2+^ release in the absence of the peptide derivatives was set as 100%. Figures represent an average of 4–5 biological replicates. E_MAX_ values are reported in **Table 1**.

We tested the ability of these molecules to potentiate 5-HT-induced 5-HT_2C_R signaling in the Ca*_i_* ^2+^ release assay (**Figure 6**; **Table 1**). Derivative **4** (E_MAX_ = 128 ± 7.4; *p* < 0.05), a head-to-tail cyclized version of **2**, results in significant potentiation of 5-HT induced Ca*_i_* ^2+^ release that is comparable with the parent peptides h3L4F and **2**. Interestingly, **5** (E_MAX_ = 107 ± 5.2), another head-to-tail cyclized peptide and its linear analog **19** (E_MAX_ = 109 ± 12), did not potentiate 5-HT-induced Ca*_i_* ^2+^ release, suggesting that these modifications may not allow the peptide to disrupt the 5-HT_2C_R-PTEN complex. The side chain-to-side chain cyclized peptide derivative **6** (E_MAX_ = 120 ± 3.0; *p* < 0.05) retained activity in the Ca*_i_* ^2+^ assay. The peptidomimetic derivative **7** (E_MAX_ = 125 ± 5.2; *p* < 0.05) also potentiated 5-HT induced Ca*_i_* ^2+^ release that is consistent with the hypothesis from the alanine scan that amino acids 3–6 are not necessary for retention of peptide activity. Furthermore, activity of **6** and **7** suggests that a β-turn conformation in the middle portion of the peptide may facilitate disruption of the 5-HT_2C_R-PTEN complex. Of note, none of the peptides alter the 5-HT potency in this assay (**Figure 6**).

**Table 1:** Effects of h3L4F and peptide 2 derivatives (1 nM) on 5-HT-induced Ca*_i_* ^2+^ release in h5-HT_2C_R-CHO cells.

Peptide Derivative ID	E_MAX_ (%5-HT)^a^	p-Value
**h-3L4F**	131 ± 6.3%	p = 0.038
**2**	118 ± 4.0%	p = 0.012
**4**	128 ± 7.4%	p = 0.020
**5**	107 ± 5.2%	p = 0.281
**19** **^b^**	109 ± 12%	p = 0.497
**6**	120 ± 3.0%	p = 0.003
**7**	125 ± 5.2%	p = 0.008

^a^Maximum 5-HT-induced Ca_i_^2+^ release (E_MAX_) in the presence of 1 nM of test peptide derivative; the screen utilized concentrations of 5-HT (vehicle, [10^-11^] to [10^-6^ M]) to establish the E_MAX_ of 5-HT in the presence of the test peptide derivative. Data represented as mean ± SEM of 4–5 biological replicates run in technical triplicates. ^b^Linear peptide after cleavage from resin was tested before the click reaction to form 5.

**Figure 7:**
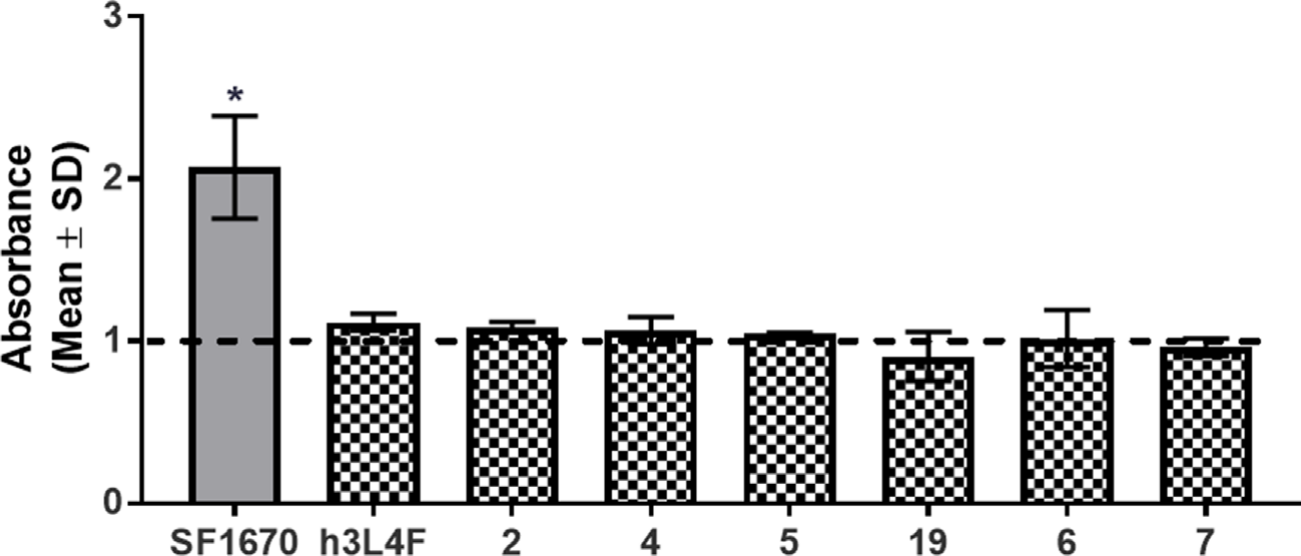
Effects of peptide **1** derivatives on PTEN lipid phosphatase activity. Absorbance is inversely correlated to amount of PIP_2_ generated in reaction of PTEN and PIP_3_ in the absence of (dashed line) or presence (checkered bars) of peptide derivatives (10 μM). Thus, elevated absorbance indicates reduced PTEN lipid phosphatase activity. PTEN inhibitor, SF1670 (200 µM; gray bar) is included as a positive control. The bars represent mean absorbance fold change over PTEN + PIP3 alone (± SD) of two independent experiments run in triplicate. *p < 0.05 vs. PTEN + PIP_3_ alone (dashed line).

PTEN is proposed to regulate 5-HT_2C_R signaling through its protein phosphatase activity (Ji et al., 2006), and thus, these peptide derivatives are not expected to disrupt the lipid phosphatase activity of PTEN. As the tumor suppressor functions of PTEN occur through its lipid phosphatase activity, we needed to ensure that our peptide derivatives do not suppress this critical PTEN function. The lipid phosphatase function of PTEN suppresses the AKT-mediated cell proliferation pathway by dephosphorylation of PI(3,4,5)P_3_ to PI(4,5)P_2_ (Maehama and Dixon, 1998), and thus, PI(4,5)P_2_ was used as an output measure for the assessment of PTEN lipid phosphatase activity in a competitive ELISA-based assay. Recombinant PTEN was incubated with PTEN inhibitor SF1670 (200 µM) or h3L4F, peptide **2** or derivatives **4**–**7** (10 µM) followed by addition of PI(3,4,5)P_3_ substrate. The absorbance, which is inversely related to the amount of generated PI(4,5)P_2_, was assessed and compared with the absorbance generated by PTEN and PI(3,4,5)P_3_ alone. There was a main effect of treatment [*F*_(8,9)_ = 8.71, *p* < 0.05; **Figure 7**]; and *a priori* comparisons show that, as expected, the PTEN inhibitor SF1670 suppresses PTEN lipid phosphatase activity. Conversely, peptides h3L4F, **2** and peptide derivatives of **2** do not suppress PTEN lipid phosphatase activity.

**Figure 8:**
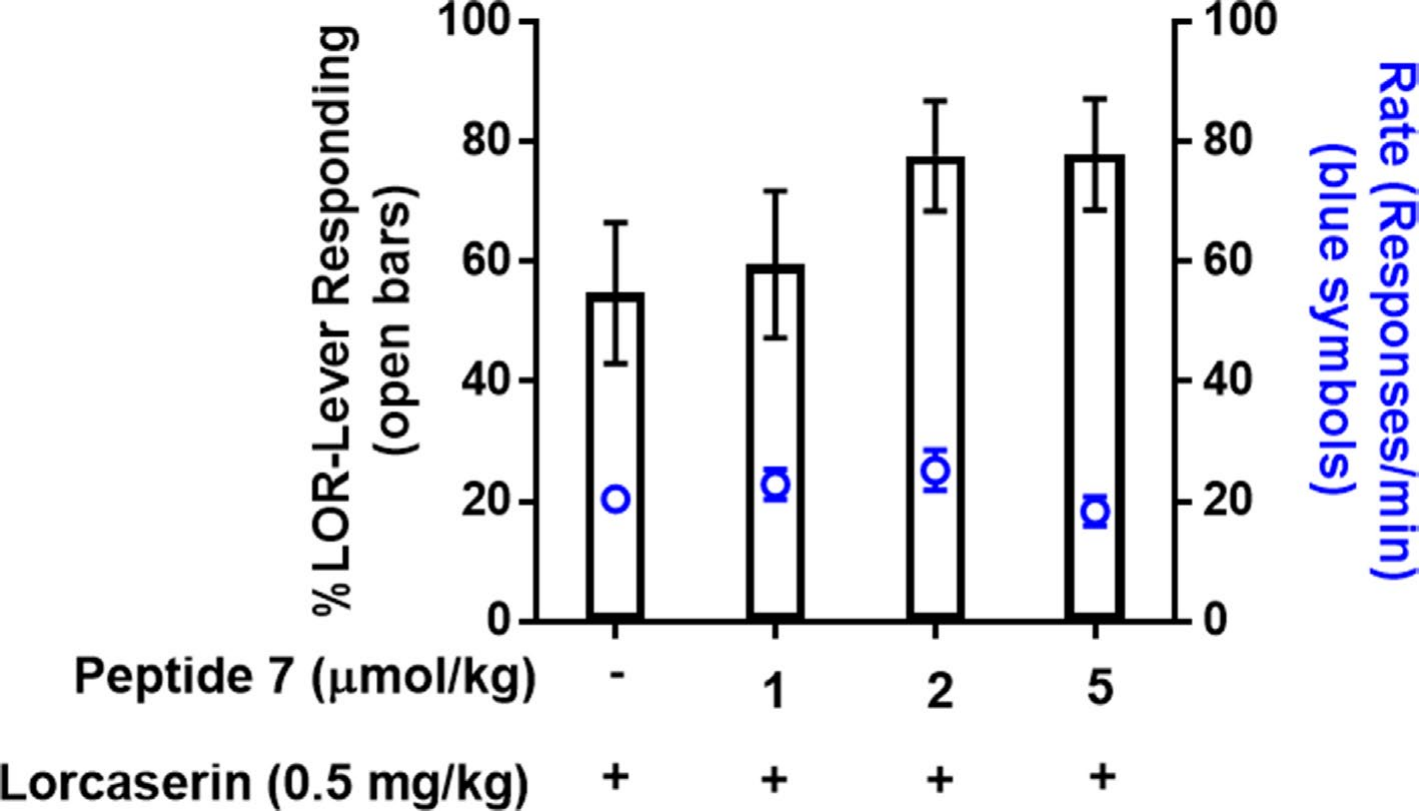
Results of combination test of lorcaserin (0.5 mg/kg) and peptide derivative 7 (1, 2, 5 µmol/kg) in rats trained to discriminate lorcaserin (n = 14). Bars represent the mean percentage of lorcaserin (LOR)-lever responding (± SEM) observed during test sessions. Blue symbols represent the rate of response in tests.

Lastly, based on the *in vitro* Ca*_i_* ^2+^ assay, peptide derivative **7** was chosen for *in vivo* assessment in lorcaserin–saline-trained drug discrimination to determine whether these modifications retain activity *in vivo*. Treatment with peptide **7** (1, 2, 5 µmol/kg) resulted in <20% drug-like responding in rats trained on lorcaserin (data not shown), suggesting that peptide **7** does not induce interoceptive cues associated with 5-HT_2C_R agonists that is consistent with TAT-r3L4F treatment in these rats (**Figure 2**). Combination tests show that peptide **7** (2, 5 µmol/kg) modestly, but not significantly, enhanced the stimulus effects of lorcaserin (0.5 mg/kg) with no change in rate (**Figure 8**). The peptide **7**-induced modest enhancement of lorcaserin stimulus effects resembles lorcaserin (0.75 mg/kg) alone, which suggests that this peptidomimetic retains activity *in vivo* consistent with potentiation of 5-HT-induced Ca*_i_* ^2+^ release in cellular assays.

## Discussion

The peptide TAT-r3L4F that disrupts the 5-HT_2C_R-PTEN interaction (Ji et al., 2006; Anastasio et al., 2013) enhanced the interoceptive cues elicited by both selective 5-HT_2C_R agonists lorcaserin and WAY163909. TAT-r 3L4F alone does not induce lorcaserin or WAY163909 stimulus generalization or reduction in response rates associated with the training drugs. These results are consistent with previously published cellular assays in which h3L4F lacks intrinsic efficacy to induce 5-HT_2C_R-associated intracellular signaling but enhances 5-HT_2C_R signaling *in vitro* and potentiates the behavioral effects of a selective 5-HT_2C_R agonist *in vivo* (Ji et al., 2006; Anastasio et al., 2013). Pretreatment with TAT-r3L4F also enhanced lorcaserin-induced suppression of the stimulus effects of cocaine. Additionally, we demonstrated that the sequence of peptide **2** can be modified to potentially increase drug-like properties and retain activity to potentiate 5-HT_2C_R signaling *in vitro* while not disrupting the lipid phosphatase activity of PTEN. Peptide **7**, the peptidomimetic derivative of peptide **2**, also modestly, but not significantly, enhanced the interoceptive cues elicited by lorcaserin. Future studies are needed to explore *in vivo* absorption, distribution, metabolism, and excretion properties to confirm that these modifications do in fact enhance the pharmacokinetic properties of the parent peptides. Together, these data suggest that disruption of the 5-HT_2C_R-PTEN complex may have positive therapeutic implications and that the generation of bioavailable disrupters with efficacy is possible.

Our data provide further evidence that TAT-r3L4F enhances 5-HT_2C_R agonist-mediated effects *in vivo*. Although the magnitude of TAT-r3L4F effects in these data is modest, TAT-r3L4F effects are reproducible in rats trained to discriminate the interoceptive cues of two selective 5-HT_2C_R agonists. These results provide further evidence that disruption of the 5-HT_2C_R-PTEN interaction may have therapeutic utility and thus provides a new target for drug discovery efforts to modulate the 5-HT_2C_R. Selective 5-HT_2C_R agonists suppress behaviors associated with drugs of abuse (for review, see Cunningham and Anastasio, 2014). One of these behaviors is the suppression of the stimulus effects of the psychostimulant cocaine (Callahan and Cunningham, 1995; Filip and Cunningham, 2002; Frankel and Cunningham, 2004). The lack of effect with TAT-r3L4F treatment in the absence of lorcaserin on cocaine drug discrimination is not unexpected given that TAT-r3L4F is not predicted to act as a 5-HT_2C_R agonist but rather potentiate the effects of 5-HT_2C_R activation. However, given that cocaine enhances the levels of endogenous 5-HT, it could be hypothesized that TAT-r3L4F treatment might suppress the stimulus effects of cocaine by enhancing endogenous 5-HT-induced 5-HT_2C_R signaling. One potential explanation for failure to see this outcome may be that the levels of endogenous 5-HT generated by cocaine exposure under the employed conditions are not sufficient for TAT-r3L4F to potentiate, which is consistent with h3L4F inability to potentiate low 5-HT concentrations in cellular assays (Anastasio et al., 2013).

Overall, the present study provides evidence that 5-HT_2C_R activity can be modulated through a protein–protein interaction. As such, this work provides the groundwork for the continued exploration of protein–protein interactions (Arkin and Wells, 2004; Wilson and Arkin, 2013; Arkin et al., 2014) that can allosterically modulate this critical receptor and other important GPCRs for new therapeutic development through mechanisms that may have enhanced clinical utility.

## Ethics Statement

Experiments were carried out in accordance with the National Institutes of Health Guide for the Care and Use of Laboratory Animals and with the approval of the Institutional Animal Care and Use Committee at University of Texas Medical Branch. The authors declare no conflicts of interest.

## Author Contributions

CS performed *in vitro* and *in vivo* assays and analyses and wrote the manuscript. H-CD, TY, and JH performed the chemical synthesis. RF performed *in vivo* assays. NA, SG, and KC conceptualized the project, oversaw experimental design/interpretation/analyses, and wrote the manuscript.

## Funding

This work was supported by National Institute on Drug Abuse grants R01 DA030977 (KC, SG), T32 DA07287 (CS), and K05 DA020087 (KC).

## Conflict of Interest Statement

The authors declare that the research was conducted in the absence of any commercial or financial relationships that could be construed as a potential conflict of interest.
